# Variants in myelin regulatory factor (*MYRF*) cause autosomal dominant and syndromic nanophthalmos in humans and retinal degeneration in mice

**DOI:** 10.1371/journal.pgen.1008130

**Published:** 2019-05-02

**Authors:** Sarah J. Garnai, Michelle L. Brinkmeier, Ben Emery, Tomas S. Aleman, Louise C. Pyle, Biliana Veleva-Rotse, Robert A. Sisk, Frank W. Rozsa, Ayse Bilge Ozel, Jun Z. Li, Sayoko E. Moroi, Steven M. Archer, Cheng-mao Lin, Sarah Sheskey, Laurel Wiinikka-Buesser, James Eadie, Jill E. Urquhart, Graeme C.M. Black, Mohammad I. Othman, Michael Boehnke, Scot A. Sullivan, Gregory L. Skuta, Hemant S. Pawar, Alexander E. Katz, Laryssa A. Huryn, Robert B. Hufnagel, The Genomic Ascertainment Cohort, Sally A. Camper, Julia E. Richards, Lev Prasov

**Affiliations:** 1 Department of Ophthalmology and Visual Sciences, W.K. Kellogg Eye Center, University of Michigan, Ann Arbor, MI, United States of America; 2 Harvard Medical School, Boston, MA, United States of America; 3 Department of Human Genetics, University of Michigan, Ann Arbor, MI, United States of America; 4 Jungers Center for Neurosciences Research, Department of Neurology, Oregon Health & Science University, Portland, OR, United States of America; 5 The Children’s Hospital of Philadelphia, Philadelphia, PA, United States of America; 6 Scheie Eye Institute, Department of Ophthalmology, Philadelphia, PA, United States of America; 7 Division of Human Genetics, Children’s Hospital of Philadelphia, Philadelphia, PA, United States of America; 8 Cincinnati Eye Institute, Cincinnati, Ohio, United States of America; 9 Molecular and Behavior Neuroscience Institute, University of Michigan, Ann Arbor, MI, United States of America; 10 Manchester Centre for Genomic Medicine, Manchester Academic Health Sciences Centre, Manchester University NHS Foundation Trust, St Mary’s Hospital, Manchester, United Kingdom; 11 Division of Evolution and Genomic Sciences, Faculty of Biology, Medicine and Health, University of Manchester, Manchester, United Kingdom; 12 Department of Biostatistics and Center for Statistical Genetics, University of Michigan, Ann Arbor, MI, United States of America; 13 Dean McGee Eye Institute, Department of Ophthalmology, University of Oklahoma, Oklahoma City, OK; 14 Medical Genomics and Metabolic Genetics Branch, National Human Genome Research Institute, National Institutes of Health, Bethesda, MD, United States of America; 15 Ophthalmic Genetics and Visual Function Branch, National Eye Institute, National Institutes of Health, Bethesda, MD, United States of America; 16 Department of Epidemiology, University of Michigan, Ann Arbor, MI, United States of America; University of Iowa, UNITED STATES

## Abstract

Nanophthalmos is a rare, potentially devastating eye condition characterized by small eyes with relatively normal anatomy, a high hyperopic refractive error, and frequent association with angle closure glaucoma and vision loss. The condition constitutes the extreme of hyperopia or farsightedness, a common refractive error that is associated with strabismus and amblyopia in children. NNO1 was the first mapped nanophthalmos locus. We used combined pooled exome sequencing and strong linkage data in the large family used to map this locus to identify a canonical splice site alteration upstream of the last exon of the gene encoding myelin regulatory factor (*MYRF* c.3376-1G>A), a membrane bound transcription factor that undergoes autoproteolytic cleavage for nuclear localization. This variant produced a stable RNA transcript, leading to a frameshift mutation p.Gly1126Valfs*31 in the C-terminus of the protein. In addition, we identified an early truncating *MYRF* frameshift mutation, c.769dupC (p.S264QfsX74), in a patient with extreme axial hyperopia and syndromic features. *Myrf* conditional knockout mice (CKO) developed depigmentation of the retinal pigment epithelium (RPE) and retinal degeneration supporting a role of this gene in retinal and RPE development. Furthermore, we demonstrated the reduced expression of *Tmem98*, another known nanophthalmos gene, in *Myrf* CKO mice, and the physical interaction of MYRF with TMEM98. Our study establishes *MYRF* as a nanophthalmos gene and uncovers a new pathway for eye growth and development.

## Author summary

Hyperopia or farsightedness is a common condition that can cause visual impairment especially in children. The extreme of this condition is called nanophthalmos, a small crowded eye in which inappropriate drainage of aqueous humor from the eye can lead to glaucoma and vision loss. We previously described a large family with inherited nanophthalmos, but the genetic defect that segregated in this family was unknown. Here, we have used a new approach combining linkage analysis and pooled sequencing to identify the genetic cause in this family. We identified a splice site mutation that causes the myelin regulatory factor (*MYRF*) gene to produce an aberrant protein. Additionally, a child with syndromic manifestations and a deleterious *MYRF* variant shared the same eye condition. Using a mouse model in which MYRF is absent from eye tissue during early development, we established a role for this transcription factor in the development of the retinal pigment epithelium and retina. We showed that MYRF interacts with and regulates expression of another membrane protein, TMEM98, which has been implicated in nanophthalmos. Our study establishes *MYRF* as a new disease gene for nanophthalmos and a regulator of eye development.

## Introduction

Uncorrected refractive error is the leading cause of moderate to severe visual impairment in the world [1]. Nanophthalmos comprises a spectrum of conditions characterized by small, structurally normal eyes and resultant high hyperopia or farsightedness. It is estimated to affect ~1% of the English population and constitutes a significant visual burden in these people [2]. Nanophthalmos can predispose individuals to vision-threatening angle closure glaucoma, increase the risk of cataract surgery complications, and is associated with amblyopia, exudative retinal and choroidal detachments, and retinal degeneration [3,4]. Hyperopia is a salient feature of specific molecular subtypes of inherited retinal degenerations [5–11]. The heritability of hyperopia is estimated through twin studies to be between 70 and 90 percent, suggesting that hyperopia is strongly influenced by genetic factors [12,13]. To date, five loci and four genes have been found in association with nanophthalmos, including sporadic, autosomal dominant and recessive forms [4]. Although large-scale genetic epidemiology studies remain to be done, known genes likely explain only a small fraction of disease burden. For example, in Chinese populations, variants in two of the most commonly mutated genes in isolated recessive nanophthalmos, *MFRP* and *PRSS56*, account for less than 6% and 8% of cases, respectively [14,15].

Thus far, genes identified in Mendelian cases of nanophthalmos have different roles in the retina (*PRSS56*, *CRB1*) [16–18] and the retinal pigment epithelium (*BEST1/VMD2*, *MFRP*) [19,20]. The precise mechanisms by which they cause defects in ocular growth are largely unknown. Species differences in ocular dimensions between mice and humans have made studying disorders of eye size more challenging [21]. Pathogenic variants in nanophthalmos genes are frequently associated with retinal degeneration [22–25], and this is often the most prominent feature in animal models [17,20,26].

Myelin regulatory factor (MYRF) is a membrane-associated homo-trimeric protein that self-cleaves to release an N-terminal domain that activates transcription [27–29]. *Myrf* has a significant role in regulating the formation and maintenance of myelination in the central nervous system (CNS) both during development and in adulthood [30–32]. Recent studies in human disease suggest that deleterious *de novo* variants in *MYRF* are associated with congenital diaphragmatic hernia, cardiac anomalies including Scimitar syndrome, urogenital anomalies, and an encephalopathy syndrome [33–36], consistent with expression in a range of tissues. However, the role of *MYRF* in ocular development has not yet been explored, and ocular phenotypes in syndromic children have not yet been evaluated.

We previously described the first large family with autosomal dominant nanophthalmos (NNO1) and mapped the locus to a 14.7 centimorgan (cM) region on chromosome 11 [37]. Here, we show that an inherited deleterious splice site mutation in *MYRF* that segregates with nanophthalmos in this family. We further demonstrate a frameshift *MYRF* variant in a child with syndromic features and an overlapping ocular phenotype. Additionally, we show that *Myrf* is expressed in ocular tissues and plays an important role in the development of the retina and RPE, and that it genetically and physically interacts with *Tmem98*, another gene implicated in nanophthalmos. Thus, we have identified a new pathway in eye growth and development and expanded the spectrum of phenotypes caused by *MYRF* mutations.

## Results

### Updated clinical phenotypes of NNO1 family

**Fig 1:**
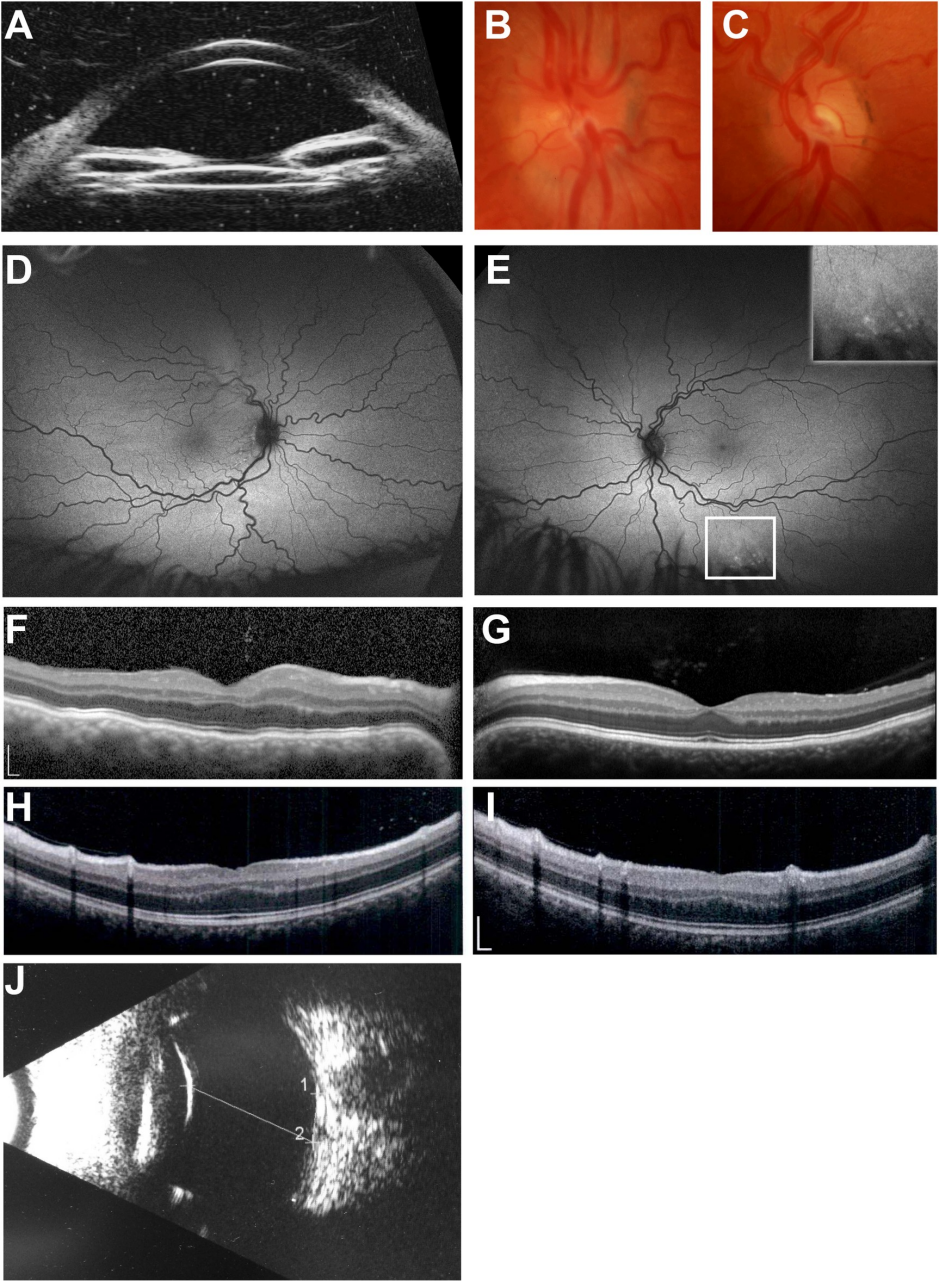
Clinical features of individuals in this study. (A-G) NNO1 family member clinical imaging. (A) Ultrasound biomicroscopy showing shallow anterior chamber and narrow angles. (B-C) Optic disc photos of the right (B) and left (C) eye showing crowded discs with vascular tortuosity. (D-E) Wide-field 200-degree Optos autofluorescence images of right (D) and left (E) showing tortuous vasculature and highlighting small area of hyperfluorescence in the left eye below the inferiotemporal arcade. (F-G) SD-OCT images of right (F) and left (G) eye showing choroidal folds in the right eye and otherwise normal foveal structure. (H-J) Sporadic nanophthalmos case clinical images. (H-I) SD-OCT of right (H) and left eye (I) eye showing mild foveal hypoplasia. (J) Bscan ultrasound showing short axial length and reduced posterior segment dimensions (line). Scale bar, 200 μm.

We first described the NNO1 family in 1998 [37]. Its phenotype is characterized by high hyperopia (+7.25 to +13.0 D) and short axial length (17.55 to 19.28 mm), with a high incidence of angle-closure glaucoma and vision loss [37]. To refine the clinical features of the affected individuals, we conducted deep ocular phenotyping in this Caucasian family to ascertain additional details of ocular structure and function. Individual IV-22 (**Fig 1**), a 31-year-old woman, had best-corrected Snellen visual acuity of 20/30 OD and 20/20 OS. Before bilateral cataract surgery for narrow angles, manifest refraction showed significant hyperopia: +9.50 sphere OD and +10.25 sphere OS. Anterior segment exam after cataract surgery showed peripherally shallow angles despite central deepening; intraocular pressure was within normal limits. Peripheral anterior synechiae concerning for plateau iris syndrome were confirmed by ultrasound biomicroscopy (**Fig 1A**). Fundus exam was notable for crowded optic discs (OD > OS) with small optic cups and vascular tortuosity (**Fig 1B and 1C**). There was a small area of non-specific stippled hyperautofluorescence in the inferior periphery of the left eye, with an otherwise normal appearing ultra-widefield short-wavelength fundus autofluorescence (**Fig 1D and 1E**). Spectral domain optical coherence tomography (SD-OCT) revealed undulations of the outer retina/RPE consistent with choroidal folds in the right eye (**Fig 1F**) and a normal foveal contour and structure in the left eye (**Fig 1G**). Electroretinography showed mildly reduced amplitudes but preserved implicit times in all waveforms under both scotopic and photopic conditions. Electro-oculogram Arden ratios were normal (2.10 and 2.18 in the right and left eye, respectively). These results were consistent with a diagnosis of nanophthalmos with no evidence of narrow angle glaucoma. There was no evidence of retinal or RPE disease with the exception of the small area of peripheral pigment mottling in the left eye and choroidal folds in the right eye.

**Fig 2:**
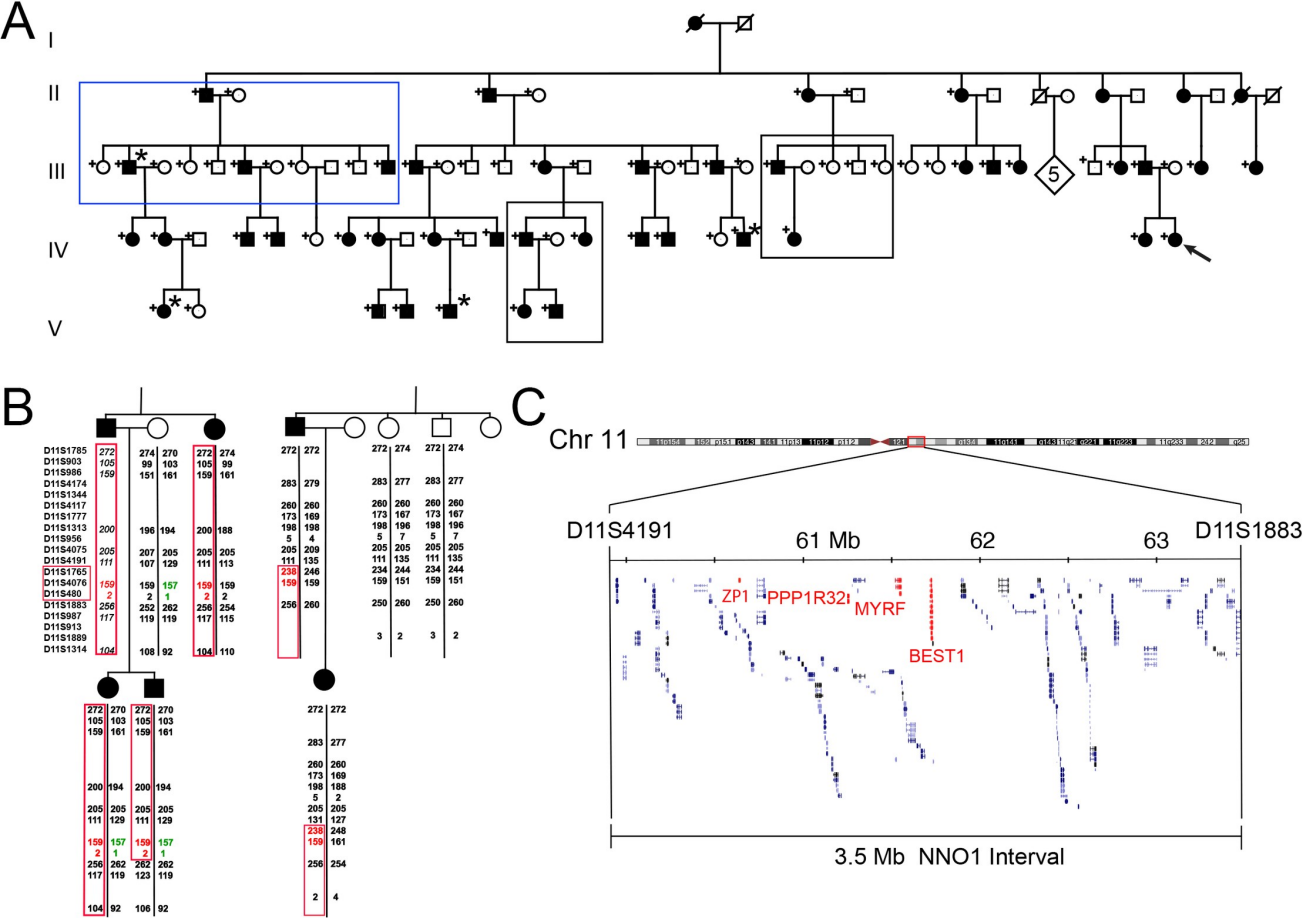
Haplotype analysis and fine mapping of NNO1 interval. (A) Updated NNO1 pedigree highlighting 4 individuals with dextrocardia (*) from different branches of the family. Blue box denotes subset of family that was subsequently chosen for segregation analysis. Black boxes indicate key regions of the pedigree with recombination events. (B) Haplotype analysis of key recombinants in the NNO1 pedigree showing minimal recombinant interval between D11S4191 and D11S1883 (highlighted in red). Disease haplotype is outlined with red boxes, and one example of shared non-disease haplotype is highlighted in green. Italics denote a deduced haplotype. (C) Genomic region encompassed by the non-recombinant interval on chromosome 11q (*MYRF*, *ZP1*, *PPP1R32*, and *BEST1* location with the interval are highlighted in red).

We conducted a medical record review of available family members. This revealed dextrocardia, a rare cardiac anomaly [38], in 4 affected and no unaffected individuals (**Fig 2A**). One of these dextrocardia patients (IV-19) was also noted to have hypoplastic right lung and pulmonary artery stenosis.

### Refinement of NNO1 interval

We originally mapped the NNO1 locus to a 14.7 cM region on chromosome 11 between microsatellite markers *D11S905* and *D11S987*, with a maximum multipoint LOD score of 6.31 at recombination fraction *θ* = 0 [37]. As a first step in identifying the underlying gene for NNO1, we screened blood samples from individuals in the family with additional microsatellite markers and reconstructed haplotypes to narrow the non-recombinant interval (**Fig 2**). This analysis refined the locus to a 3.5 MB region between *D11S4191* and *D11S1883*, which contains 135 genes (**Fig 2)**. The locus included the *VMD2/BEST1* gene previously implicated in nanophthalmos that encodes a calcium-activated chloride channel associated with nanophthalmos and four types of retinal dystrophy [19,24,39–45]. Sanger sequencing of all of the coding elements of *BEST1*, including 50–100 bp of flanking intronic sequences, revealed no pathogenic variants (**S1 Fig**). Long range PCR was used to exclude exonic deletions of *BEST1* (**S1 Fig**). Sequencing of these fragments, with identification of SNPs in the fragments, confirmed that both copies of *BEST1* were being amplified using this method. Molecular analysis was then extended to include introns and untranslated regions (UTR1/2) of *BEST1*. This identified a range of common variants and known SNPs, but no ultra-rare variants that were considered either to be novel or potentially pathogenic. Furthermore, observation of normal Arden ratios on the EOG in the nanophthalmos individual suggest an alternative to *BEST1* as the cause of disease, as individuals carrying pathogenic mutations in *BEST1*-associated nanophthalmos have an abnormal EOG Arden ratio [24].

### Pooled exome sequencing and variant calling

**Fig 3:**
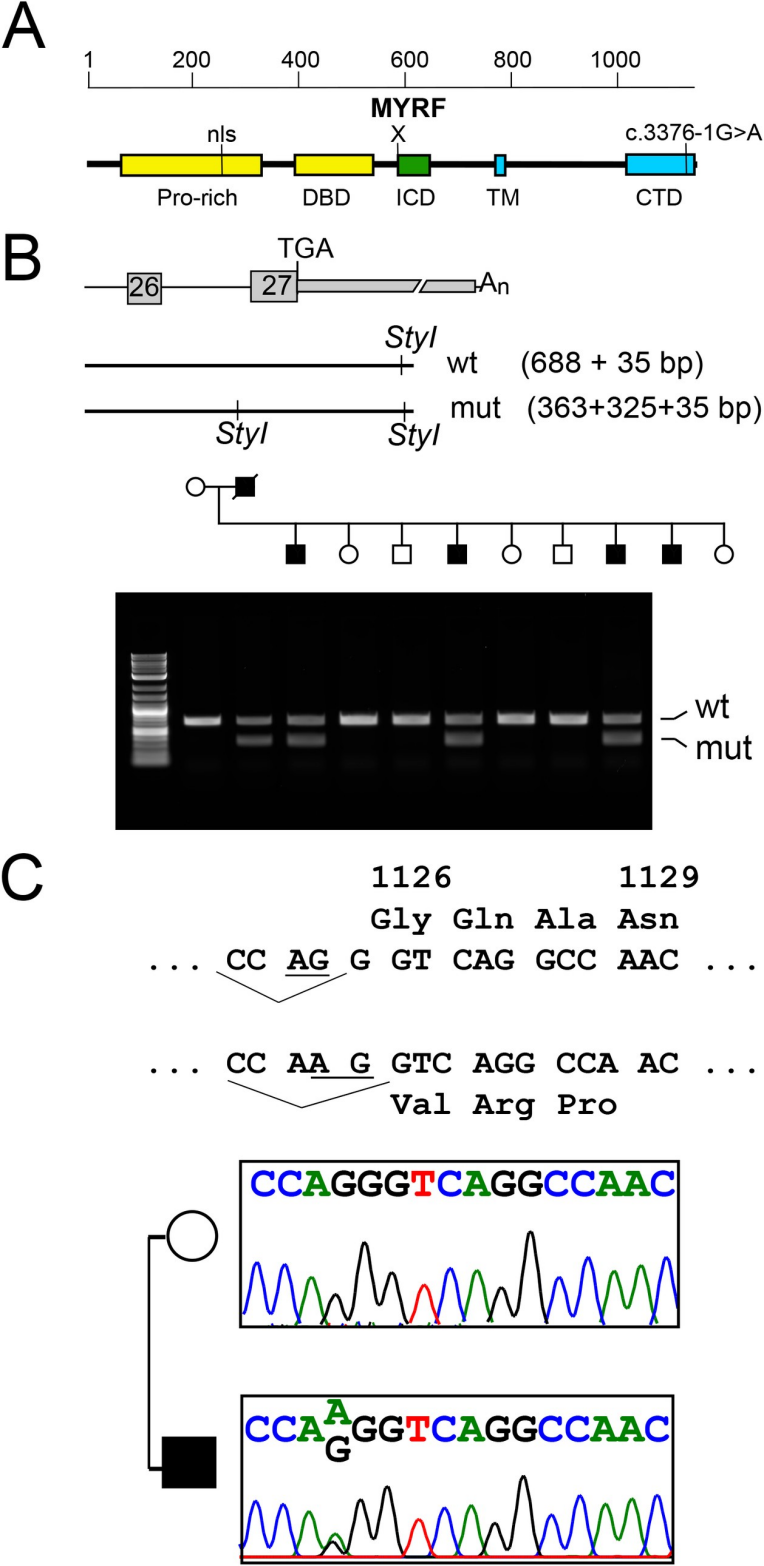
The *MYRF* c.3376-1G>A variant co-segregates with the nanophthalmos within the NNO1 family. (A) Schematic diagram of MYRF protein and functional domains. (B) Schematic and agarose gel electrophoresis for *Sty*I restriction digest used to confirm variant segregation in NNO1 family within one large nuclear family branch. (C) Sequence of normal and variant MYRF with splice acceptor site (underlined) and predicted amino acids. Sequencing chromatograms confirming heterozygous c.3376-1G>A mutation. Pro-rich, proline-rich domain; nls, nuclear localization sequence; DBD, DNA binding domain; ICD, intramolecular chaperone domain; TM, transmembrane domain; CTD, C-terminal domain.

Given the large number of candidate genes within the interval, we employed a new approach to identify candidate variants, combining pooled whole exome sequencing together with our linkage data. We separately pooled DNA samples of 26 affected individuals and 13 unaffected individuals for whole exome sequencing and subtractive filtering (**S2 Fig**). The unaffected pool was selected to maximize the representation of the non-disease haplotypes found in affected individuals. We expected the difference in minor allele fraction in the affected vs. unaffected pool (ΔMAF) to be between 0.4 and 0.6 for variants within the linkage interval, but we used a more conservative cutoff of 0.1 to improve the sensitivity of detecting variants. We performed whole exome sequencing as described in the methods and identified 1144 variants within the linkage interval. After filtering for rare nonsynonymous coding exonic, splice junction, or microRNA variants with ΔMAF>0.10, three candidate variants remained (**S1 Table**). No coding or non-coding variants were found in *BEST1*. As expected for variants on the disease haplotype located within the linkage interval, all of these variants segregated perfectly with the disease phenotype (**Fig 3, S4 Fig**). We excluded two of these candidate variants, in *ZP1* and *PPP1R32*, on the basis of the presence of homozygotes within the Genome Aggregation Database (gnomAD) [46] and allele frequency (0.2%) being higher than the rate of severe nanophthalmos in the general population (**S1 Table**). Furthermore, *ZP1* has undetectable expression in human ocular tissues by RT-PCR and expression sequence tags (ESTs) in the Unigene database (National Center for Biotechnology Information), and PPP1R32 loss-of-function mice had no clear ocular phenotype in the International Mouse Phenotyping Consortium Database [47].

The remaining candidate variant was a conserved splice acceptor mutation in the myelin regulatory factor gene (NM_001127392.2; *MYRF* c.3376-1G>A). This variant was absent in 246126 alleles in gnomAD, highly conserved in all vertebrate species, and in the top 0.1% of damaging variants by CADD score [48]. Evaluation of evolutionary constraint showed that *MYRF* was significantly constrained against missense and loss-of-function (LoF) variation (pLI = 1.00), while *PPR1R32* was not (**S3 Fig**). Taken together, these findings suggested that *MYRF* c.3376-1G>A was the most promising candidate mutation.

### Identifying ocular phenotypes in patients with *MYRF* variants and prevalence of *MYRF* variants in nanophthalmos

To validate *MYRF* as a nanophthalmos gene, we identified an additional, unrelated syndromic patient with a deleterious variant in *MYRF* and evaluated him for ocular phenotypes. The 8-year-old male subject was initially seen in the comprehensive ophthalmology clinic at age 4. His visual acuity was 20/30 in each eye at that time with a +9.00 sphere cycloplegic refraction and an otherwise unremarkable ocular exam. A focused follow-up exam at age 8 revealed +7.00 sphere manifest refraction and B-scan ultrasonography demonstrated normal ocular structures in the setting of short eyes (17 mm axial length in each eye, **Fig 1J**). Fundus exam appeared normal. SD-OCT showed a flattened foveal contour with a visible inner nuclear layer (INL) at the foveal center and mild increase in outer nuclear layer (ONL) thickness and outer segment lengthening, in line with grade I foveal hypoplasia based on published criteria [49] (**Fig 1H and 1I**). These findings supported a diagnosis of nanophthalmos. Additionally, this child had systemic findings of mitral valve prolapse, unilateral cryptorchidism, and micropenis, consistent with *MYRF* spectrum disorders [33,34,36]. Clinical exome sequencing revealed a c.769dupC (p.S264QfsX74) variant in *MYRF*. Chromosomal microarray was normal. The patient’s mother was a mosaic carrier of this variant, was asymptomatic, and had a normal ocular exam. This variant was absent from the gnomAD database and expected to be deleterious as the RNA species would be expected to undergo nonsense-mediated decay given the early truncation of the protein prior to the DNA-binding, transmembrane, and the intramolecular chaperone assembly (ICA) domains.

To evaluate the prevalence of *MYRF* mutations in high hyperopia and nanophthalmos, we systematically screened 60 independent cases of high hyperopia and nanophthalmos. These included both sporadic and inherited cases, where nanophthalmos was defined as axial length <21 mm in the smaller eye and high hyperopia was defined as cycloplegic or manifest refraction with spherical equivalent >+5.50 D. We identified several rare variants, but none are likely to be causative based on higher allele frequencies in gnomAD controls than the expected prevalence of nanophthalmos in the population (**S2 Table**).

These results lend further support that loss-of-function variants in *MYRF* lead to nanophthalmos, which can present in syndromic or predominantly isolated forms. To determine whether other rare variants in *MYRF* were associated with nanophthalmos or high hyperopia, we identified individuals in The Genomic Ascertainment Cohort (TGAC), whose exome data were available from the ClinSeq project [50]. We selected three individuals with variants in *MYRF* (**S3 Table**) that were predicted to be deleterious by a high CADD score and very rare (<0.1%) allele frequency for ocular phenotyping. None of the carriers of these variants had high hyperopia or nanophthalmos, as determined by our clinical criteria (see methods). No loss-of-function variants were identified in the TGAC cohort, and it is not unusual for *in silico* damaging variants of unknown significance to be tolerated [51].

### *MYRF* variant disrupts splicing causing alteration of the C-terminus

**Fig 4:**
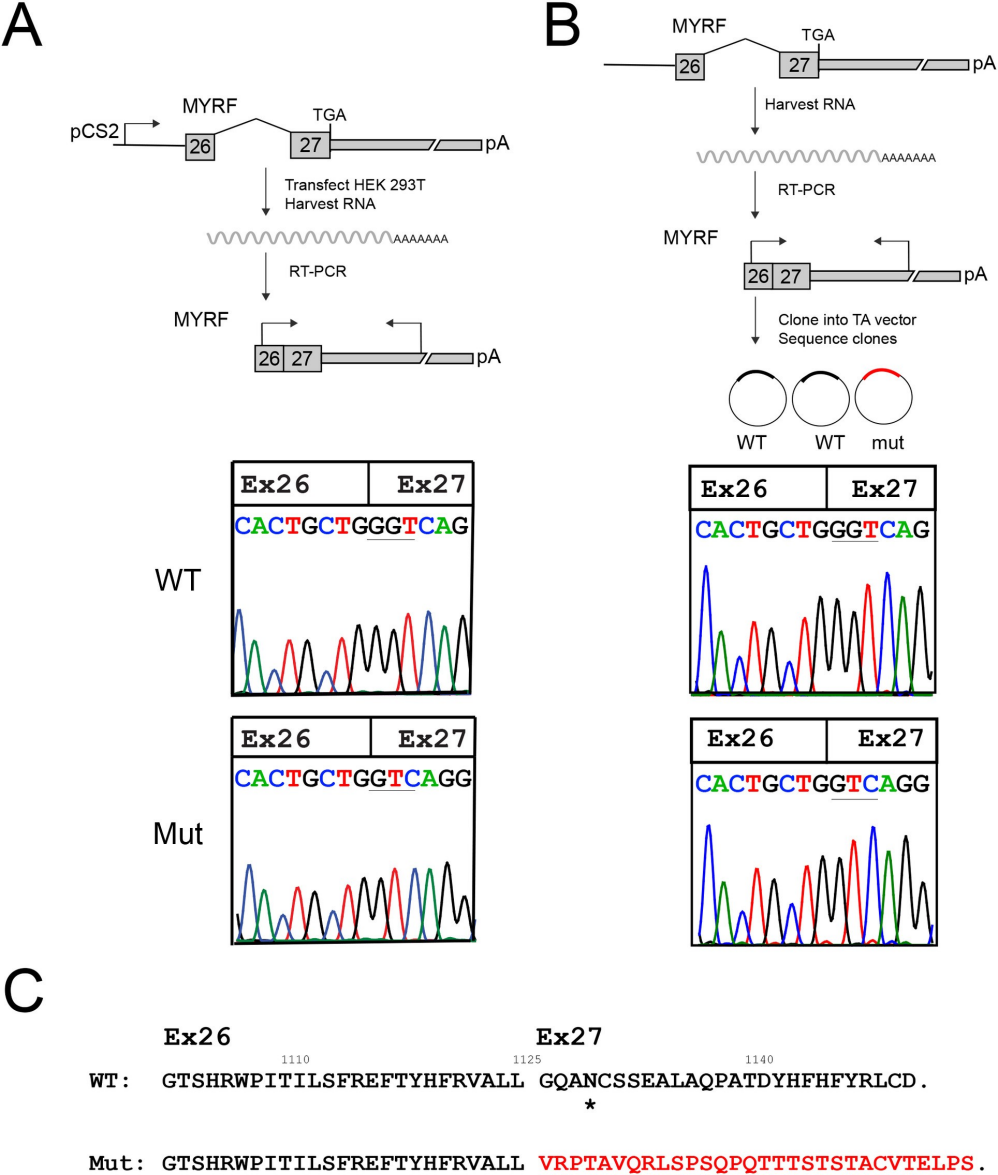
*MYRF* c.3376-1G>A variant disrupts mRNA splicing and produces a stable RNA species. (A) Minigene splicing assay. Schematic diagram showing the design of the minigene assay, and sample chromatograms showing the wild-type and mutant cDNA species isolated from this assay. A single splice product was generated for each form, wild-type (WT) and mutant (Mut) RNA. The splice product for the mutant form uses a splice acceptor site one base pair downstream of the original splice site and creates a frameshift (see **Fig 3C**). (B) RT-PCR from individual IV-22 blood RNA. Schematic diagram of the experiment is shown with sequence chromatograms from representative cloned RT-PCR products demonstrated. The mutant clones comprised 42% (3/7) of the spliced RNA species. (C) Predicted effect of splice site mutation on C-terminal amino acid sequence, showing frameshift and replacement of the final 26 amino acids with 30 different amino acids. A putative glycosylation site is marked with a *.

*MYRF* is a 27-exon gene that encodes a homo-trimeric membrane protein that autoproteolytically cleaves and translocates to the nucleus to regulate transcription [27–30,52]. The *MYRF* c.3376-1G>A variant is predicted to disrupt a conserved splice accepter upstream of the last exon (**Fig 4**), and this residue is highly conserved in all vertebrate species. Using the Alternative Splice Site Predictor tool (ASSP) [53], we predicted that the G>A change would result in loss of the splice acceptor site for the last exon, and formation of a new splice acceptor site 1 basepair (bp) downstream of the original site.

To test this hypothesis, we first designed a mini-gene assay to test the effect of the loss of the canonical splice acceptor site in MYRF. We cloned the human wild-type and mutant genomic sequence from *MYRF* exon 26 through the polyadenylation site in exon 27 into the CS2 expression vector [54], which uses a CMV promoter to ubiquitously drive gene expression. Subsequently, we transfected each of these constructs into HEK293T cells, isolated RNA, and then reverse-transcribed and sequenced the resulting cDNA. The wild-type construct showed consistent splicing from exon 26 to exon 27 of *MYRF* (**Fig 4A**). The *MYRF* c.3376-1G>A mutation, however, abolished this splice acceptor site and led to use of a splice site 1 bp downstream as predicted.

To further confirm that the aberrant *MYRF* RNA is generated *in vivo*, we collected blood from individual IV-22 and isolated RNA to look at the relative abundance of RNA species in this individual. Given the low level of *MYRF* expression in blood, we cloned reverse-transcribed and PCR amplified sequences spanning exons 26 to 27 to better quantify the relative abundance of spliced transcripts *in vivo*. The mutant splice product was generated in 3/7 clones, consistent with the predicted 50% from monoallelic expression (**Fig 4B**). These results are consistent with the suggestion that the *MYRF* c.3376-1G>A variant generates a stable mRNA product, and that the relative expression of wild-type and mutant RNA species is roughly equivalent. The splice alteration is predicted to lead to a 1bp frameshift in the coding sequence of the C-terminus resulting in replacement of the final 26 amino acids of the protein with a different 30 amino acids, and disruption of a putative glycosylation site (**Fig 4C**).

### MYRF expression pattern

**Fig 5:**
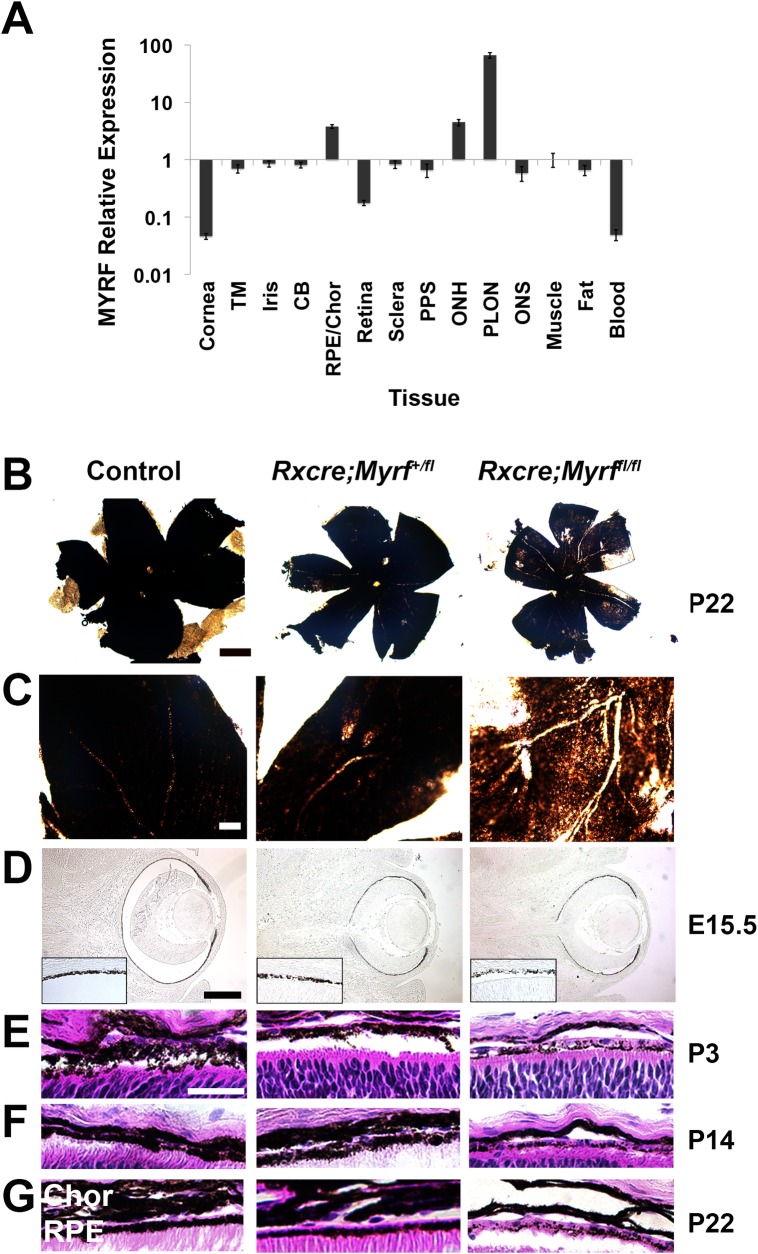
*MYRF* is expressed in the RPE and loss of MYRF in mouse leads to pigmentary change in the mouse RPE. (A) qRT-PCR showing relative expression of *MYRF* in human ocular and adnexal tissue. *MYRF* expression is normalized to *GAPDH* and reported as expression level relative to extraocular muscle. (B-C) Low-magnification (B) and high magnification (C) RPE flatmounts from P22 control (*Myrf*^+/fl^ or *Myrf*^*fl/fl*^) and *Myrf* heterozygous and homozygous conditional knockout mice (*RxCre;Myrf*^*+/fl*^ and *RxCre;Myrf*^*fl/fl*^) showing patchy areas of hypopigmentation in the *RxCre;Myrf^fl/fl^* mice. (D-F) H&E histology of RPE from the above mice at E15.5 (D), P3 (E), P14 (F), and P22 (G) showing early loss of RPE pigmentation (D) in *RxCre;Myrf*^*fl/fl*^ mice, which persists after retinal histogenesis is complete (G). TM, trabecular meshwork; CB, ciliary body, Chor, choroid; PPS, peripapillary sclera; ONH, optic nerve head; PLON, post-laminar optic nerve. Scale bar, 500 μm in B, 100 μm in C, 50 μm in D, 25 μm in E.

To define the role of MYRF in the pathogenesis of nanophthalmos, we first systematically evaluated RNA expression in human ocular and adnexal tissues. We identified highest levels of *MYRF* expression in the RPE/choroid and in the optic nerve, with low levels in other ocular tissues relative to extraocular muscle (**Fig 5**). These results are consistent with the known role of MYRF in myelination of the optic nerve and relative expression data based on RNAseq experiments in human ocular tissues [55]. A similar pattern of expression was observed in mouse ocular tissues with a 99.5±1.8 fold greater expression in RPE as compared to retina by qRT-PCR. Immunofluorescence staining of mouse retinal sections for MYRF using the validated N-terminal antibody [28,56] showed consistent expression in the RPE in human and mouse tissue; however, it is likely that nonspecific cross-reactivity to retinal tissue complicates the interpretation of these results (**S5 Fig**).

### Loss of MYRF causes RPE loss and early retinal degeneration, but minimal effect on eye size

**Fig 6:**
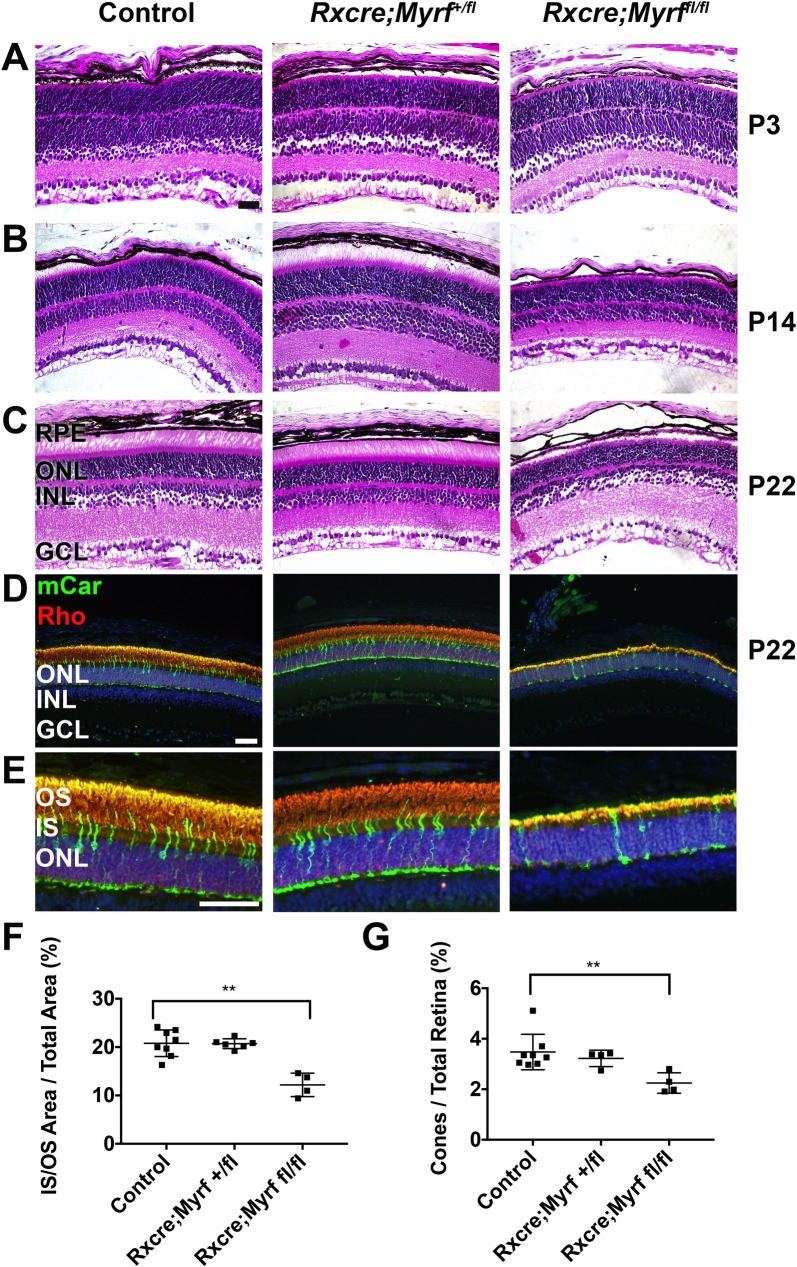
Loss of MYRF leads to early retinal degeneration in mice. (A-C) H&E histology of P3 (A), P14 (B), and P22 (C) control (*Myrf*^+/fl^ or *Myrf*^*fl/fl*^) or *Myrf* heterozygous and homozygous conditional knockout mice (*RxCre;Myrf*^*+/fl*^ and *RxCre;Myrf*^*fl/fl*^). *RxCre;Myrf*^*fl/fl*^ retinas have shortened inner and outer segments, but retinal structure during development is preserved. *RxCre;Myrf*^*+/fl*^ are structurally indistinguishable from control. (D-E) Low magnification (D) and high magnification (E) images of photoreceptor immunolabeling in P22 animals. Mouse cone arrestin (mCar, green) and rhodopsin (Rho, red) and DAPI (blue) were used to mark cones, rods, and nuclei, respectively. (F) Quantitative analysis of inner/outer segment area compared to total retinal area. (G) Quantitative analysis of cone fraction compared to fraction of total retinal cells. There is a significant decrease in IS/OS area and cone fraction in *RxCre;Myrf*^*fl/fl*^ retinas. n = 8 eyes, 6 animals (control); n = 6 eyes, 3 animals (*RxCre;Myrf*^*+/fl*^), n = 4 eyes, 4 animals (*RxCre;Myrf*^*fl/fl*^). Mean±standard deviation are plotted along with each individual eye data point. **, p<0.01. Scale bar, 50μm.

To understand the pathophysiology of *MYRF* mutations causing eye disease, we generated a loss-of-function mouse model in the early eye field. Specifically, we used a Cre driver that begins expression in the early developing retina and RPE at embryonic day (E) 10.5 (*RxCre*) [57] to delete exon 8 of *Myrf* (*Myrf*^*fl*^), leading to a non-functional protein in these cells [30]. Gross examination of eyes and RPE flatmounts from *RxCre;Myrf*^*fl/fl*^ mice at postnatal day (P) 22 revealed patchy loss of RPE pigmentation in all mutant mouse eyes compared to heterozygous (*RxCre;Myrf*^*+/fl*^) and wild-type littermates (**Fig 5B and 5C**). Gross analysis of axial length in enucleated eyes, measured as central cornea to optic nerve distance, did not reveal any substantial or statistically significant differences in eye size among genotypes at P22 or gross differences at other time points (**S6 Fig**): 2.93±0.07 mm for control (n = 7 animals, 14 eyes), 2.95±0.09 mm *RxCre;Myrf*^*+/fl*^ (n = 3 animals, 6 eyes), and 2.95±0.07 mm *RxCre;Myrf*^*fl/fl*^ mice (n = 3 animals, 6 eyes). To detect more subtle biometric changes, we conducted anterior chamber and vitreous chamber analysis using SD-OCT in a separate cohort of animals. Measurement of anterior chamber depth did not reveal any statistically significant difference among the genotypes (**S6 Fig**): 0.31±0.02 mm for control (n = 8 eyes from 4 mice), 0.30±0.02 mm for *RxCre;Myrf*^*+/fl*^ (n = 5 eyes from 3 mice), 0.31±0.03 mm for *RxCre;Myrf*^*fl/fl*^ (n = 11 eyes from 6 mice), one-way ANOVA F = 0.29, p = 0.79 (**S6H Fig**). Likewise, there was a minimal statistical or biological differences on the vitreous chamber depth: 0.69±0.02 for control (n = 8 eyes from 4 mice), 0.67±0.006 mm for *RxCre;Myrf*^*+/fl*^ (n = 6 eyes from 3 mice), 0.67±0.02 mm for *RxCre;Myrf*^*fl/fl*^ (n = 12 eyes from 6 mice), one-way ANOVA F = 2.058, p = 0.15 (**S6G Fig**). Given that *Myrf* is absent in early development in *RxCre;Myrf*^*fl/fl*^ mice, we evaluated the histologic appearance at various developmental time points. We noted that patchy loss of RPE pigmentation is present as early as E15.5 (**Fig 5D, S7 Fig**). Histologic analysis confirmed segmental losses of RPE granular pigment and thinning of the choroid with otherwise maintained cells (**Fig 5E–5G**). To investigate whether the patchy appearance of pigmentation was due to mosaic deletion from the *RxCre* driver, we crossed the *RxCre* mice with the *Rosa26floxYFP* reporter line [58]. Our results revealed that there was constitutional expression of YFP throughout RPE and weaker, but uniform expression in the retina (**S8 Fig**). In *RxCre;Myrf*^*fl/fl*^*;R26floxYFP* mice, RPE cells were likewise uniformly YFP+, suggesting that variability in pigmentation is not due to selective loss of MYRF in these areas. Additionally, we generated *Myrf*^*+/-*^and *RxCre;Myrf*^*fl/-*^ mice to determine whether Cre deletion efficiency plays a role in the severity and uniformity of the RPE phenotype. *RxCre;Myrf*^*fl/-*^ showed comparable and patchy RPE pigmentation loss and similar retinal phenotype to *RxCre;Myrf*^*fl/fl*^ (**S9 Fig, Fig 6C**). Taken together, these results suggest that the variability and changes in pigment deposition through development were less likely to be due to differential deletion of the *Myrf* floxed allele or issues with Cre recombination efficiency.

Given the disparate ocular anatomy in the mouse versus human eye and the difficulty in observing small differences in mouse eye size, we next evaluated the effects of *Myrf* deletion on the structure of the outer retina. Histologic analysis *of RxCre;Myrf*^*fl/fl*^ revealed shortened photoreceptor inner and outer segments (IS and OS) and thinning of the ONL (65% of wild-type, p = 0.001) especially overlying depigmented areas (**Fig 6**). Histologic analysis at post-natal day (P) 3 did not reveal any differences in retinal structure (**Fig 6A**). *RxCre;Myrf*^*fl/fl*^ mice eyes had shortened outer segments by P22 (**Fig 6B and 6C**). To quantify the loss of photoreceptors, we identified the fraction of cone and rod photoreceptors by immunostaining against rhodopsin (rhodopsin) or cone-arrestin (cones) (**Fig 6D and 6E**). Our results showed a statistically significant loss of IS/OS area compared to total retinal area (12±2% vs. 21±3%, p = 0.001, **S4 Table, Fig 6F**) in *RxCre;Myrf*^*fl/fl*^ mice compared to controls and preferential loss of cone photoreceptors (2.3±0.4% vs. 3.5±0.7% of total retina cells, p = 0.004). Furthermore, we see a greater effect on IS/OS area than on ONL area (20±2% vs. 24±3% in *RxCre;Myrf*^*fl/fl*^ vs. control, p = 0.01, **S4 Table**) We did not see any significant differences in any of these measures between control and *RxCre;Myrf*^*+/fl*^ eyes. We also evaluated measurements of the retinal thickness, and the number of cells in each cell layer. These analyses did not show any significant differences in the number or fraction of cells in the retinal ganglion cell layer (**S4 Table**). A small, but significant difference was noted in overall retinal area in the *RxCre;Myrf*^*fl/fl*^ vs. control mice (**S4 Table**). This was consistent with measurements of retinal thickness obtained by SD-OCT at P22 in a separate cohort of mice (**S6F Fig**): 0.232±0.006 mm for control (n = 8 eyes from 4 mice); 0.220 ±0.003 mm for *RxCre;Myrf*^*+/fl*^ (n = 6 eyes from 3 mice), p = 0.0003; 0.211±0.005 mm for *RxCre;Myrf*^*fl/fl*^ (n = 12 eyes from 6 mice), p = 1.6x10^-6^ vs. control and p = 0.00013 vs. *RxCre;Myrf*^*+/fl*^.

**Fig 7:**
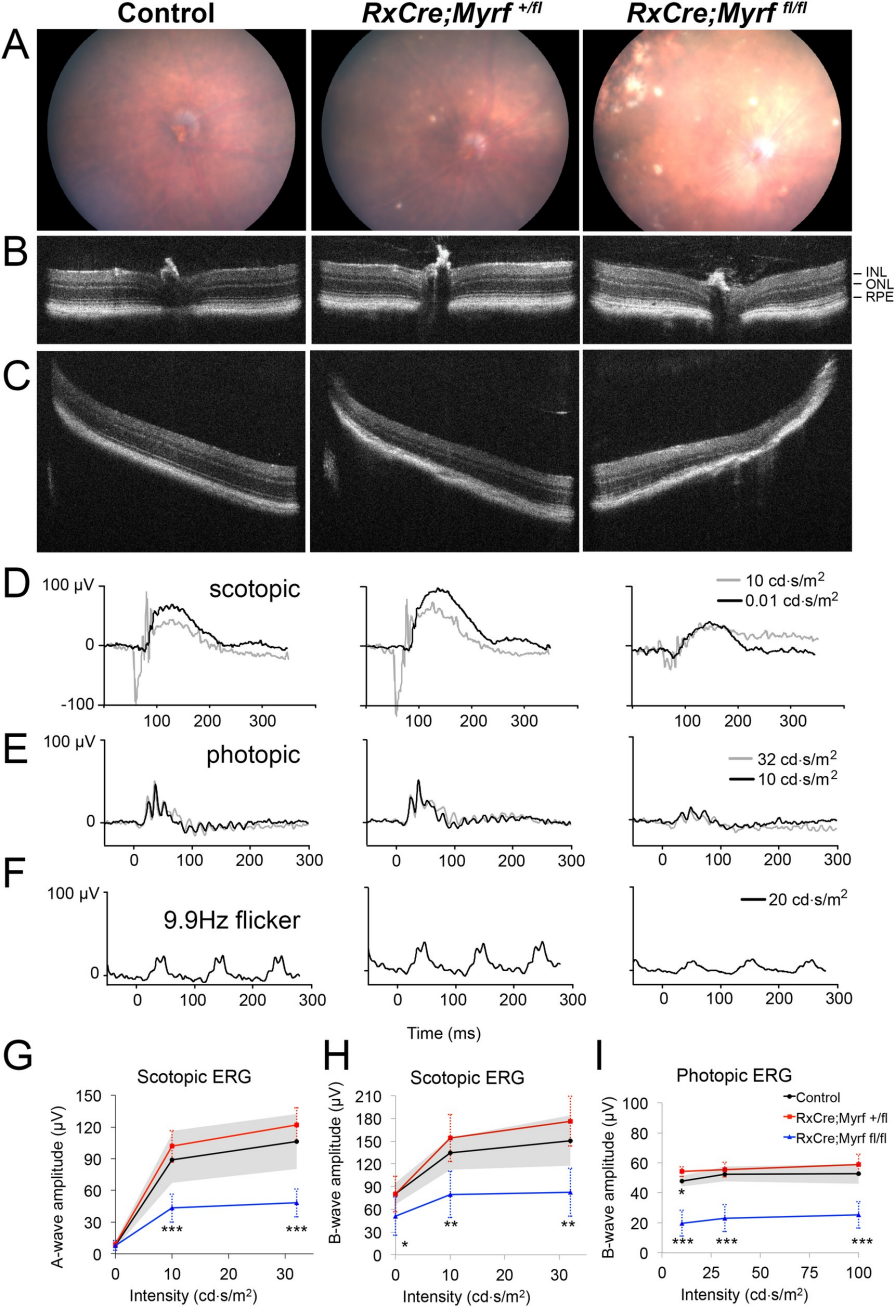
Loss of MYRF leads to global retinal dysfunction and patchy outer retinal atrophy. (A-C) Representative central color fundus photos (A) and central (B) and peripheral (C) SD-OCT from 10-month old eyes from control (*Myrf*^fl/fl^ or *Myrf*^*+/fl*^), *RxCre;Myrf*^*+/fl*^ and *RxCre;Myrf*^*fl/fl*^ mice. Patchy areas of atrophy with RPE pigment changes are seen most prominently in *RxCre; Myrf* ^*fl/fl*^ eyes (A), and correspond to outer retinal and RPE loss on OCT (C) with relatively preserved peripapillary retina (B). (D-I) Electroretinography of 10-month old control (*Myrf*^*fl/fl*^ or *Myrf*^*+/fl*^), *RxCre;Myrf*^*+/fl*^ and *RxCre;Myrf*^*fl/fl*^ mice. (D-F) Representative electroretinogram traces from 10-month old mice under scotopic (D), photopic (E), or photopic 9.9 Hz flicker conditions with the noted intensity stimuli. Both scotopic and photopic responses are diminished in *RxCre;Myrf*^*fl/fl*^ eyes compared to controls. (G-I) Scotopic a-wave (G), scotopic B-wave, and photopic B-wave amplitudes and comparison statistics across varying intensity stimuli. Error bars indicate standard deviation and are noted by the shaded grey for the control group. Summary statistics for comparisons of *RxCre;Myrf*^*+/fl*^ and *RxCre;Myrf*^*fl/fl*^ to control eyes are shown with stars. In *RxCre;Myrf*^*fl/fl*^, scotopic a-wave ERG amplitudes are reduced by 45–50% and B-wave amplitudes are reduced 35–45%, while photopic B-wave amplitudes are reduced 50–60%. Control n = 4 eyes from 2 animals, *RxCre;Myrf*^*+/fl*^ n = 4 eyes from 2 animals, *RxCre;Myrf*^*fl/fl*^ n = 12 eyes from 6 animals. Mean±standard deviation. ***, *p* < 0.001; **, *p* < 0.01; * *p* < 0.05.

To further define the retinal phenotype of *RxCre;Myrf*^*fl/fl*^ mice and confirm the retinal degeneration phenotype, we next pursued *in vivo* structural and functional testing using a separate cohort from our histologic analysis. Using 10-month-old *RxCre;Myrf*^*fl/fl*^ and age-matched heterozygous and wild-type controls, we evaluated the appearance of the retina by fundus photography and SD-OCT. Photography showed patchy areas of RPE and retinal atrophy, with variable pigment deposition in *RxCre;Myrf*^*fl/fl*^, but not control mice (**Fig 7A**). *RxCre;Myrf*^*+/fl*^ mice had a few smaller, but similar looking lesions. On SD-OCT, there was evidence of outer retinal disruption in *RxCre;Myrf*^*fl/fl*^ with severe loss of the ONL and RPE in affected areas in the retinal periphery and milder changes centrally (**Fig 7B and 7C**). *RxCre;Myrf*^*+/fl*^ eyes were grossly normal appearing on SD-OCT (**Fig 7B and 7C**). To evaluate the functional consequences of these effects, we conducted electroretinograms (ERGs) on these mice. We saw significantly diminished scotopic and photopic responses in *RxCre*;*Myrf*^*fl/fl*^ eyes compared to control eyes at all stimulus intensities (**Fig 7D–7I, S5 Table**). In *RxCre;Myrf*^*fl/fl*^, scotopic a-wave ERG amplitudes were reduced by 45–50% and B-wave amplitudes are reduced 35–45%, while photopic B-wave amplitudes were reduced 50–60% (**Fig 7G–7I**). Photopic flicker peak amplitudes were likewise reduced by 43%. Taken together, our findings strongly support that *RxCre;Myrf*^*fl/fl*^ eyes undergo retinal degeneration, with both rod and cone pathways affected. It remains to be determined whether the *RxCre;Myrf*^*+/fl*^ mice have a more subtle, late onset retinal degeneration, but we did not observe any histologic changes in the early postnatal period or ERG or SD-OCT findings in 10-month-old mice (**Figs 6 and 7**).

### MYRF has a physical and genetic interaction with TMEM98

**Fig 8:**
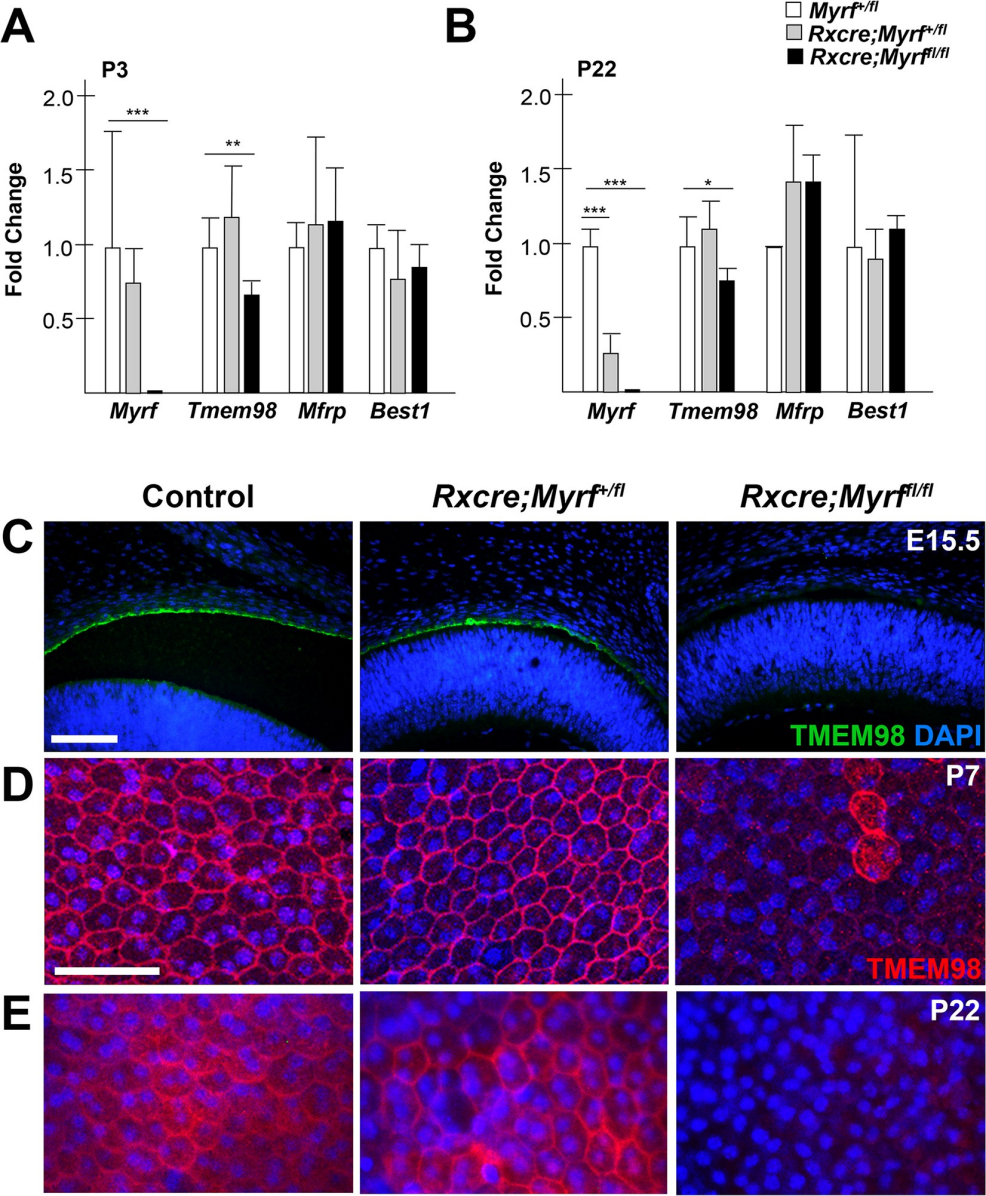
TMEM98 is genetically downstream of MYRF. (A-B) Taqman qRT-PCR analysis of RPE expressed genes important for nanophthalmos (*Myrf*, *Best1*, *Mfrp*, *Tmem98*) in *Myrf*^*+/fl*^, *RxCre;Myrf*^*+/fl*^ and *RxCre;Myrf*^*fl/fl*^ eyes at P3 (A) and P22 (B). There is significantly reduced expression of *Tmem98* in *RxCre;Myrf*^*fl/fl*^ compared to wild-type, but comparable levels of expression of *Mfrp* and *Best1*. (C) TMEM98 staining in embryonic (E15.5) mouse eyes showing loss of TMEM98 staining in *RxCre;Myrf*^*fl/fl*^ compared to controls. (D-E) Mouse RPE flat mounts from P7 (D) and P22 (E) eyes showing decreased TMEM98 staining (red) and altered localization in *RxCre;Myrf*^*fl/fl*^ compared to controls. DAPI (blue) is used to counterstain nuclei. ***, *p* < 0.001; **, *p* < 0.01; * *p* < 0.05.

Given that mouse MYRF functions as a transcription factor [28,29,59] and is present in fetal RPE, we hypothesized that it might regulate transcription of other RPE-expressed genes associated with nanophthalmos in humans. To investigate this, we evaluated expression of *Best1*, *Mfrp*, *Myrf*, and *Tmem98* by qRT-PCR in developing (P7) and mature eyes (P22) from *RxCre;Myrf*^*fl/fl*^, *RxCre;Myrf*^*+/fl*^, and wild-type littermate controls. As expected, *Myrf* RNA levels were nearly absent in *RxCre;Myrf*^*fl/f*l^ mutants and reduced in *RxCre;Myrf*^*+/fl*^ mice (**Fig 8A**). Consistent with our previous findings that MYRF has a binding site just upstream of the transcriptional start site of the *Tmem98* gene in oligodendrocytes [28], we noted that *Tmem98* mRNA levels were significantly reduced in eyes from *RxCre;Myrf*^*fl/fl*^ mice as compared to heterozygous and wild-type controls (**Fig 8A**). There was no difference in mRNA level between the genotypes for *Mfrp* or *Best1*.

Given the reduction in *Tmem98* mRNA in mice lacking *Myrf*, we evaluated the effect at the protein level by immunostaining in sections and RPE flatmounts at different developmental stages. Our results demonstrated a profound reduction in TMEM98 immunostaining with a small fraction of cells with diffuse cellular localization pattern, possibly mosaicism-related (**Fig 8 and S10 Fig**). These findings are seen early in development (E15.5, **Fig 8C**) and persist through later developmental stages (**Fig 8D and 8E**). In contrast, we saw no distinguishable difference in staining for MFRP (**S11 Fig**).

**Fig 9:**
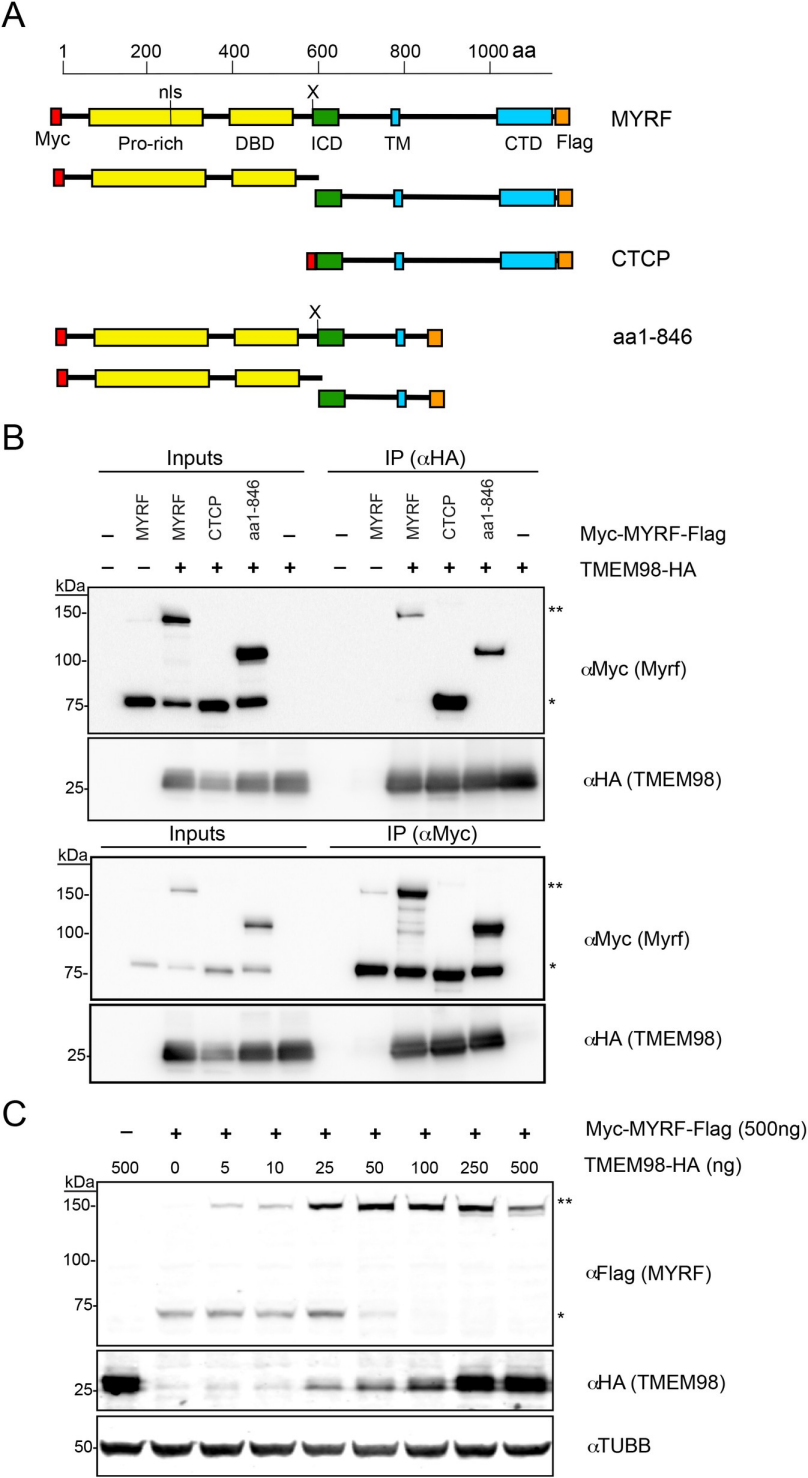
MYRF physically interacts with TMEM98. (A) Schematic diagram of N-terminal Myc-tagged and C-terminal HA-tagged MYRF constructs used for co-immunoprecipitation experiments. Below each construct, the observed cleavage products are shown. Note that only the N-terminal product can be detected with anti-Myc, and the C-terminal product with anti-FLAG, and all constructs are expected to be cleaved except the one corresponding to the already-cleaved C-terminal cleavage product. (B) Co-immunoprecipitation (co-IP) experiments with HA-tagged TMEM98 and Myc-tagged MYRF constructs in HEK293T cells. Extracts from HEK293T cells were transfected with the indicated constructs, and then immunoprecipitated with anti-HA (top) or anti-Myc (bottom). Western blots against Myc or HA are shown for each immunoprecipitation experiment. HA-TMEM98 immunoprecipitates with either full-length Myc-MYRF, the uncleaved form of a Myc-MYRF^1-846^ construct or a Myc-C-terminal-cleavage-product (aa587-1139), mapping the region of interaction to MYRF^587-846^. (C) Western blots showing stabilization of uncleaved MYRF in a dose dependent manner by increasing levels of TMEM98. Extracts from HEK293T cells were transfected with myc-MYRF-FLAG and increasing doses of HA-TMEM98, and subsequently blotted against C-terminal FLAG tag (MYRF), N-terminal HA tag (TMEM98), and loading control β-III tubulin (TUBB3). MYRF, full-length MYRF; CTCP, C-terminal cleavage product; aa1-846, MYRF truncated construct (amino acids 1–846); ** corresponds to full-length MYRF band, * corresponds to cleavage product band (N-terminal in B, C-terminal in C). The N-terminal and C-terminal cleavage products run at a similar size due to post-translational modification.

As feedback loops are a common regulatory mechanism in development [60], we hypothesized that TMEM98 may function in a feedback loop with MYRF. To evaluate this possibility, we conducted co-immunoprecipitation experiments with HA-tagged TMEM98 and myc-tagged MYRF (**Fig 9**) in HEK-293T cells to look for a direct interaction between these proteins. We were able to pull down HA-TMEM98 with the full-length myc-MYRF and vice versa, suggesting that these two proteins interact directly (**Fig 9B**). We observed that TMEM98 preferentially interacts with the un-cleaved form of MYRF; no band for the MYRF N-terminal cleavage product was observed with the HA-TMEM98 immunoprecipitation, suggesting that TMEM98 does not interact with the N-terminal cleavage product that comprises the functional transcription factor. TMEM98 also stabilized the un-cleaved form of MYRF in a dose-dependent manner (**Fig 9C**). To further define the interaction site of these two proteins, we generated a truncating mutant containing the first 846 amino acids of MYRF (deleting much of the ER-lumenal component of the protein), and a mutant consisting of only the C-terminal cleavage product. Both of these truncated proteins retained their ability to pull down TMEM98 (**Fig 9**), suggesting that the region in MYRF that is critical for this interaction is downstream of the cleavage site in the intramolecular chaperone or transmembrane domains, between amino acids 586 and 846.

## Discussion

NNO1 was the first locus implicated in the pathogenesis of nanophthalmos [37]. We present multiple lines of evidence to implicate a deleterious variant in *MYRF* as the underlying cause of nanophthalmos in the family that we used to map the NNO1 locus. First, *MYRF* c.3376-1G>A alters mRNA splicing leading to a significant alteration in the C-terminus of MYRF. This change is absent in large exome and genome databases including gnomAD, 1000 Genomes, and dbSNP [46,61,62]. The base is highly conserved, and *MYRF* is strongly constrained against loss of function variants (pLI = 1.00) [46] (**S3 Fig**). Second, MYRF is expressed in the RPE in mice and humans, much like several other regulators of eye size [19,63,64]. Third, no other compelling variants in other genes were identified within the linkage region. *BEST1* and other candidates were excluded as a potential cause by variant analysis, direct sequencing, and further patient phenotyping, including a normal EOG. Fourth, loss of mouse *Myrf* leads to RPE depigmentation and retinal degeneration, a similar phenotype as that caused by mouse models of other nanophthalmos genes such as *Mfrp* [20]. Fifth, *MYRF* interacts with *TMEM98*, a gene recently implicated in nanophthalmos in 3 families [63,64], at both regulatory and direct protein-protein levels. Sixth, a syndromic patient with an *MYRF* loss-of-function variant also has small eyes and high hyperopia. Taken together, we have provided strong evidence for a causal link between *MYRF* and high hyperopia/nanophthalmos.

### *MYRF* variants and human disease

Since the discovery of the membrane-associated *Myrf* transcription factor [28–30], much has been learned about its role in human disease, and its structure and function. *De novo* variants have been implicated in a new syndrome involving congenital diaphragmatic hernia, cardiac defects, and urogenital anomalies [33,34,36], and in an encephalopathy syndrome [35]. The mutational spectrum of these variants predominantly includes truncating mutations that would be predicted to undergo nonsense-mediated decay or missense mutations within the DNA binding and intramolecular chaperone auto-processing (ICA) domains [28,29,59]. Both of the functional domains are critical to processing and protein function, and are conserved in lower order species including the slime mold *Dictyostelium* [59,65]. The splice site variant in the NNO1 family is vertically transmitted, affects the C-terminus of the protein, and results in a predominantly ocular phenotype. Although significant advances have been made in elucidating the role of the N-terminal domains of *Myrf*, including the transcriptional targets and the cleavage process [28,29,56,59,66], the role of the C-terminus remains unclear. Interestingly, much of the C-terminus of MYRF is absent in lower eukaryotes [65], suggesting that this domain likely developed disparate functions in higher eukaryotes.

Our large family with nanophthalmos represents an expansion in phenotype of that seen in the newly described MYRF-associated syndrome [33,34,36]. Patients in our large family have a severe ocular phenotype, but few have systemic features. Four affected individuals have reported dextrocardia, a very uncommon finding in the general population [38]. Scimitar syndrome, an anomalous venous return syndrome associated with *MYRF* mutations [33], may be misdiagnosed as isolated dextrocardia [67]. As such, dextrocardia in these individuals may represent a milder form of the cardiac anomalies present in syndromic patients carrying *MYRF* loss of function variants.

The differences in pathogenesis of *MYRF* variants and in ocular and systemic phenotypes could be explained in several ways. First, MYRF may be a dosage sensitive transcription factor with ocular tissues being more sensitive than cardiac and urogenital tissues. In this model, the C-terminal alleles would be hypomorphic causing mild disruption in protein function, while early truncating or non-functional alleles would lead to haploinsufficiency in all affected tissues. The variable penetrance of the dextrocardia phenotype may be consistent with this model. However, data from animal model studies favors an alternative model, as *Myrf* heterozygous mice (*Myrf*^*+/-*^ *and Myrf*^*fl/-*^) are viable and have normal Mendelian segregation ratios (40 *Myrf*^*+/-*^ vs. 38 *Myrf*^*+/+*^ pups, χ^2^ p = 0.82). This would be contrary to what would be expected with the severe human phenotype [33,34,36]. Furthermore, *RxCre;Myrf*^*+/fl*^ and *Myrf*^*+/-*^ mice have a minimal ocular phenotype (**Figs 5–7, S9 Fig**).

Alternatively, *MYRF* variants may lead to dominant negative effects in protein function, through their incorporation into trimers. This effect may alter protein-protein interactions, cleavage, or ability to activate transcription. Differential effects may be observed in different tissues, such that the eye is more sensitive to changes in the C-terminus of the protein. Future work will be necessary to determine which of these mechanisms explains the ocular and systemic phenotypes in these patients.

Our results support the need for comprehensive ophthalmic evaluation and potential intervention in patients with *MYRF* mutations, even if they are presumed asymptomatic, as subnormal vision may be overlooked in severe systemic disorders. Uncorrected high hyperopia can be associated with strabismus and amblyopia in early childhood, which may cause permanent vision loss [68]. Proper early spectacle correction along with surveillance and treatment of ocular motility abnormalities may help to improve final visual outcomes [69,70] and consequently quality of life. Nanophthalmos may also predispose to angle closure glaucoma early in life [71,72]. The risks of this visually disastrous complication may be mitigated with early intervention by peripheral iridotomy or lens extraction, and avoidance of triggering medications [73]. On the other end of the syndromic spectrum, cardiac evaluation in NNO1 family members and other cases with *MYRF* mutations may also be warranted, as Scimitar syndrome and other anomalous pulmonary venous return phenotypes are often asymptomatic, but can be associated with dyspnea, fatigue, and recurrent respiratory infections [74].

### Towards a new pathway for nanophthalmos: MYRF interaction with TMEM98

High hyperopia is strongly heritable and known genes likely account for a small fraction of explained cases [12–15]. Our analysis of unrelated high hyperopia and nanophthalmos probands suggests that variants in *MYRF* are an uncommon monogenic cause of these conditions. Thus, nanophthalmos is highly genetically heterogenous, and a load of deleterious variants in multiple genes may influence the severity of the phenotype. It remains to be determined whether other rare or common variants in *MYRF* contribute to high hyperopia.

The discovery of a membrane-associated transcription factor in the pathogenesis of nanophthalmos suggests that downstream targets and interacting partners may serve as excellent candidate genes for high hyperopia and refractive error in general. In oligodendrocytes, SOX10 and MYRF have been shown to activate dual-specificity phosphatase (*Dusp15*), which in part contributes to oligodendrocyte differentiation [66]. Intriguingly, *Dusp15* is also expressed in fetal RPE [55], warranting a further exploration of this pathway in ocular development.

**Fig 10:**
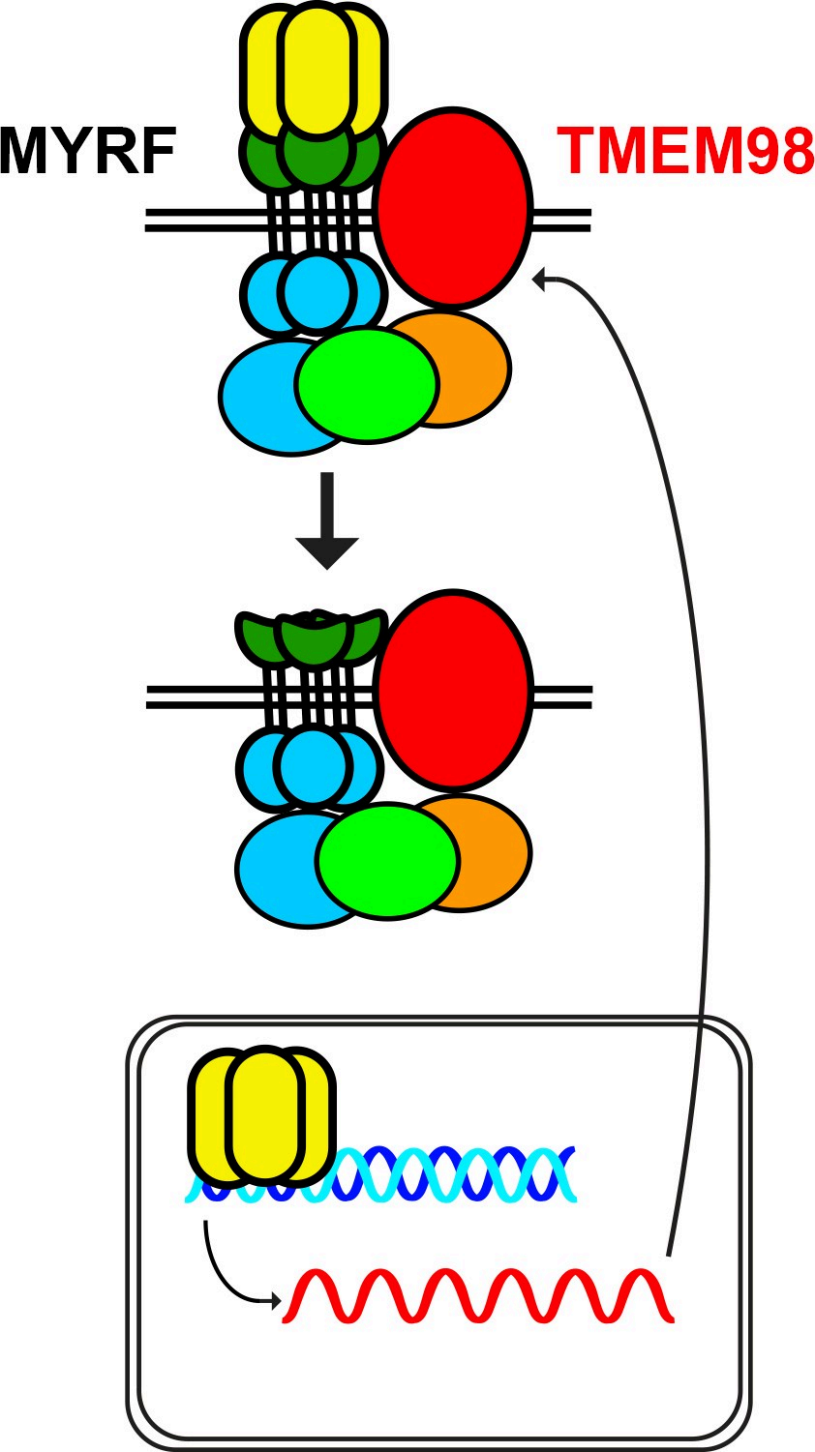
Model of MYRF and TMEM98 function. MYRF interacts with TMEM98 and other factors, as yet unidentified, and upon cleavage activates transcription of specific downstream genes, including TMEM98, in various tissues including RPE. MYRF domains including the N-terminal (yellow), intramolecular chaperone (green), and C-terminal (blue) domains are highlighted.

*TMEM98* variants have been implicated in autosomal dominant nanophthalmos in three families [63,64], but little is known about the function of this protein in the eye. In our report, we demonstrate a genetic and physical interaction between MYRF and TMEM98 (**Figs 8 and 9**). These results are consistent with a recent report demonstrating an interaction of between TMEM98 and MYRF in oligodendrocytes in mice and also an interaction between the *C*. *elegans* orthologs TMEM98 and MYRF-1 [75]. Both of these proteins are membrane-associated [28,29,75]. Using deletion constructs, Huang et al. narrowed the interaction domain to the region between the transmembrane binding and the C-terminal domains (amino acids 765–1003 in mouse MYRF). Taken together with these findings, our results further narrow the interaction interval to residues 765–849, suggesting that the transmembrane domain and surrounding sequence are critical for interaction. The NNO1 *MYRF* splice site mutation, however, is downstream of this critical region, suggesting that it is unlikely that disruption of the TMEM98 interaction leads to pathogenesis in this family. Similar to Huang et al., we observed that overexpression of TMEM98 stabilizes the uncleaved form of MYRF (**Fig 9** and [75]). In our mouse model, *Tmem98* mRNA and protein expression is significantly reduced in *Myrf* CKO mice (**Fig 8**), suggesting that *Tmem98* is also a downstream target of *Myrf* through direct transcriptional activation, indirectly in the RPE developmental pathway, or both. Consistent with this, the *Tmem98* gene is a direct MYRF target in oligodendrocytes [28,75]. Although this suggests a negative feedback loop, it remains unclear whether the stabilization of the ER-bound MYRF precursor by TMEM98 represents a physiologic function or an artifact of over-expression systems. It is likely that other co-factors interact with TMEM98 and MYRF, and these may lead to tissue specific effects in function (**Fig 10**). Further work will be necessary to elucidate this novel pathway that controls RPE development in mice and likely controls eye growth in humans.

### Differences in biometry and ocular development in mouse and man

Ocular dimensions and proportions differ significantly in humans and mice, with a much higher lens volume in mouse ocular tissues [21]. Additionally, the average adult human eye is 23.4 mm [76,77], while the average adult C57BL/6J mouse eye measures only 3.2 mm [78]. Therefore, detecting small differences in mouse ocular size is difficult. It is not unexpected for a mouse model of a human nanophthalmos gene mutation to have a relatively normal eye size. Loss-of-function of *Mfrp* (rd6 mouse) [20] and *Crb1* [17] predominantly feature normal eye size with retinal degeneration in mouse models and yet cause nanophthalmos in humans [25,79]. *Best1* knock-out and knock-in mice show normal histology with no evidence of retinal degeneration or change in eye size [80,81], while individuals with *BEST1* mutations can have 4 distinct retinal dystrophies [24,80]. Similarly, nanophthalmos-associated human variants in *TMEM98* [63,64] when knocked-in to the *Tmem98* locus in mice cause retinal white spots and retinal folds, but no appreciable difference in ocular size [82]. Even in mouse models where an ocular biometric phenotype was detected, the difference is small. For instance, the difference in ocular size between wild-type and mutant mice was 4% for *Prss56* on a C57BL/6J background [16], and less than 5% for *Mfrp* [83]. In humans, in contrast, the difference in axial length for nanophthalmos patients from their unaffected family members and the general population is roughly 25–30% [84,85]. Furthermore, genetic background has a strong influence on eye size in mice [86], and can be a modifier of the effect of deleterious mutations [16,18].

As such, it is not surprising that the most prominent ocular phenotype of *RxCre;Myrf*^*fl/fl*^ mice is a retinal degeneration and RPE pigmentation defect rather than a substantial ocular size difference, much like in the *Mfrp* rd6 mice [20] and *Crb1* rd8 mice [17]. Indeed, we observe a substantial retinal degeneration that involves loss of both cone and rod function, detected with both histology and ERG testing (**Figs 6 and 7**). We see no significant difference on the ganglion cell layer thickness or cell density in P22 mice, but future work will be necessary to rule out the development of glaucoma in this animal model, and to determine whether the retinal ganglion cell density is truly unaffected, given that displaced amacrine cells comprise 60% of the GCL [87].

It remains possible that *Myrf* modulates eye size in mice and we have not fully excluded this in our analysis. Our sample sizes would be underpowered to detect small differences, and enucleation based methods of eye size determination can be prone to fluctuation. Additionally, as ocular growth continues linearly in mice past sexual maturity [88], it remains possible that a larger difference in eye size would be observed in older animals. Nonetheless, we have shown that there is no large effect on eye size in our animal model. Further work with isogenic strains and more sensitive measurement techniques, including OCT and refractive measurements, will be necessary to determine this in the future [78,89].

### Combined linkage and pooled exome approach for gene discovery

Our results highlight the efficacy of a linkage and pooled sequencing approach in the context of gene discovery. Combining linkage and exome or genome sequencing approaches provides a powerful filter for next-generation sequencing data, increasing the likelihood of identifying genetic variants by targeting the search [90]. Similarly, pooled exome sequencing provides a cost-effective method for gene discovery [91]. Here, we have coupled linkage with a pooled exome sequencing approach for gene discovery in a large family. This approach has several advantages. First, it allows for disease haplotype enrichment, which leads to an enhanced signal to noise ratio in exome data. By having a large pooled sample of affected and unaffected individuals, unaffected haplotypes are depleted from the variant pool, leading to a much smaller pool of variants to analyze. Variants present within one or a small number of individuals are diluted in the pool. Even prior to population-based allele frequency and *in silico* protein damaging effects, only 9 candidate variants remained within the linkage interval. Second, signal averaging minimizes the impact of mis-phenotyping sequenced patients. Given the report of the aggregate genotype, a single mis-phenotyped person would contribute only 1/N of the sequence reads, where N is the number of individuals. This would have virtually no effect on our ability to call a heterozygous variant. In traditional exome-based approaches, these variants may be initially filtered, as there is expectation of shared variants among all affected individuals within a family and absence of these variants in unaffected individuals [92]. Third, this method is very cost-effective, requiring only a single lane of sequencing for 2 pooled samples with high coverage depth. Fourth, by pooling of multiple affected individuals, this method is unable to return individual secondary findings, such as the ACMG 59 [93], to patients. Thus, it bypasses the patient concerns regarding these findings that would otherwise need to be addressed, especially when studies are conducted on a research basis [94].

We have used this novel pooled-exome and linkage-based approach to uncover the role of *MYRF* in the pathogenesis of nanophthalmos. We have further uncovered a role for MYRF in the early development of the RPE, and a regulatory and physical interaction with TMEM98 another RPE expressed gene implicated in nanophthalmos. These results have primed future work to elucidate the developmental pathways controlling eye growth, which will be important for developing novel therapies for counteracting the extremes of refractive error.

## Materials and methods

### Ethics statement

This study was carried out according to the standards Declaration of Helsinki and the Common Rule of the United States Federal Government (46CFR45) under protocols approved by the Institutional Review Boards of the participating institutions and by the Office of Human Research Subject Protection at the National Institutes of Health. Mouse studies were approved by the Institutional Animal Care and Use Committee at the University of Michigan.

### Human subjects

We described the 5-generation family of European and Native American ancestry in the original linkage study [37]. Additional clinical phenotyping was performed on the individual IV-22, as detailed above. Additional medical record review was done for all available patients and family members to evaluate for systemic associations with cardiac, neurologic, and urologic disorders.

Forty-eight unrelated probands with nanophthalmos/high hyperopia and twelve children with high hyperopia were screened for mutations in all of the *MYRF* coding exons as described below. Nanophthalmos was defined as an axial length of less than 21 mm in at least one eye with no more than 2 mm difference in axial length, as measured by optical biometry by IOLMaster (Zeiss, Oberkochen, Germany) or Lenstar (Haag-Streit, Köniz, Switzerland). Several of these individuals were previously described in a large case series [95], and 6 probands had other documented affected family members. High hyperopia was defined as at least +5.50 spherical equivalent on cycloplegic or manifest refraction in the more affected eye.

Three patients from the ClinSeq project [50] as part of The Genomic Ascertainment Cohort were identified that carry potentially deleterious variants in *MYRF* (**S2 Table**). The individuals were evaluated for ocular phenotypes by standard ocular exam including slit lamp biomicroscopy and ocular biometry at the National Institutes of Health Clinical Center.

### Haplotype analysis, *BEST1* exclusion, pooled whole exome sequencing and variant analysis

DNA from the NNO1 family was genotyped for 22 microsatellite markers within the 14.7 cM linkage interval. Haplotypes were reconstructed using familial relationships, and recombinant individuals were identified. For each individual, a haplotype was assigned to each chromosome within the linkage region.

Haplotype analysis using STR loci (**S1 Fig**) within the *BEST1/VMD2* locus was done on two affected individuals (V-7, IV-20). Analysis of the entire coding sequence and 50–100 of surrounding intronic sequence was done via Sanger sequencing using methods and primer sequences previously described [24]. Untranslated (UTR1/2) regions were amplified using the primers in **S1 Fig**. Long range PCR using two sets of primers (LR1/2 and LR3/4) covering coding exons 2–6 and 7–10 was done to exclude exonic deletions of *BEST1*.

A DNA sample pool was prepared from 26 affected individuals. A separate pool of 13 unaffected individuals was used for subtraction, with the goal of enriching for the disease haplotype. Subsequently, DNA libraries of ~375 bp were constructed, the exome was enriched using SeqCap EZ Human Exome Library v.3.0 (Roche, Pleasanton, CA, USA), and paired-end sequencing was conducted using the Illumina HighSeq 1000 platform (Illumina, San Diego, CA, USA). Sequencing was done to achieve >100X coverage for the genes in this region. Paired-end reads were aligned with the Burrows-Wheeler Aligner [96], and recalibrated and realigned using the Genome Analysis Toolkit (GATK) [97]. Variants were called using the UnifiedGenotyper in GATK, filtered for the non-recombinant interval using VCFTools [98], and annotated using SeattleSeq [99].

### Mutation screening and segregation analysis

To verify transmission of mutations through the family, DNA from one large branch (**Fig 2A**) was amplified using the primers listed in **S6 Table, S7 Table** for *PPP1R32*, *ZP1*, and *MYRF*, which encompassed the identified variants. For segregation analysis of the *MYRF* variants, amplified PCR products were subsequently digested with *StyI* to generate restriction fragment length polymorphisms (RFLPs). These were then separated on a 1.2% agarose gel to identify the genotypes. Similar segregation analysis was done for *PPP1R32* and *ZP1* using different RFLPs (**S4 Fig**).

*PPP1R32*, *ZP1*, and *MYRF* coding regions were amplified from genomic DNA extracted from whole blood or mouthwash samples using the DNeasy Blood kit (Qiagen, Germantown, MD, USA). PCR primers and conditions are listed in **S6 Table**. PCR products were diluted or cleaned using exo-SAP (Thermo Fisher Scientific, Waltham, MA, USA) or a PCR purification kit (Qiagen) and sequenced on an ABI3730 DNA Analyzer (Applied Biosystems, Thermo Fisher Scientific) at the University of Michigan DNA sequencing core. All variants were compared to high-quality sequence reads in the Exome Aggregation Consortium (ExAC) or gnomAD databases [46]. All coordinates in this report are based on NCBI reference genome build 37.1 (hg19).

### *MYRF* mini-gene splicing analysis and splicing evaluation in NNO1 patient

A region of *MYRF* containing sequences from Exon 26 through the polyadenylation site was amplified from NNO1 patient DNA (IV-12) using the primers and PCR conditions in **S5 Table** and Expand HiFidelity polymerase (Roche). The PCR product was purified using the QIAspin PCR purification kit (Qiagen), and subcloned into pCS2 vector, which drives expression using the simian cytomegalovirus IE94 enhancer/promoter. A construct bearing the wild-type copy and one with the patient variant were used for subsequent analyses.

Transfections were performed into HEK293T cells using FuGENE 6 reagent according to manufacturer’s instructions (Promega, Madison, WI, USA). Cells were harvested 48 hours after transfection. RNA was extracted using Trizol reagent (Invitrogen, Thermo Fisher Scientific) and treated with DNaseI, and reverse-transcribed using the High-Capacity cDNA Reverse Transcription Kit (Applied Biosystems, Thermo Fisher Scientific) according to manufacturer’s instructions. The region surrounding the splice site was amplified using the primers and conditions listed in **S5 Table**, purified with QIAspin PCR purification kit and sequenced.

For splicing assessment from patient RNA, DNaseI-treated total RNA from patient IV-22 was transcribed into cDNA as above. The sequence spanning the splice site was amplified using the primers and conditions listed in **S5 Table**. Amplicons were cloned into the pGEMTeasy TA cloning vector system (Promega) and DNA was isolated from clones and sequenced using the amplification primers at the University of Michigan DNA Sequencing Core.

### Quantitative RT-PCR

Ocular and adnexal tissues including cornea, trabecular meshwork, ciliary body, iris, extraocular muscle, periorbital fat, trabecular meshwork, retina, RPE/choroid, sclera, peripapillary sclera, optic nerve head, post-laminar optic nerve, and optic nerve sheath were dissected from a 71-year-old Caucasian female. Total RNA was prepared from most of the dissected eye tissues using the RNeasy kit (Qiagen). RNA from iris, ciliary body, and RPE was prepared using an RNAqueous-rPCR kit (Invitrogen) to eliminate co-purification of pigment. cDNA was generated by reverse transcription (RT) using the High-Capacity cDNA Reverse Transcription Kit (Applied Biosystems) according to manufacturer’s instructions. Quantitative RT-PCRs were performed using the Taqman system with inventoried probes (Applied Biosystems), and analyzed on a CFX384 Touch Real-Time PCR Detection System (Bio-Rad, Hercules, CA, USA). Critical cycle threshold levels were normalized to GAPDH controls run in parallel. Fold activity was calculated using the ddCt method [100] and reported relative to extraocular muscle tissue.

For animal experiments, eye tissues were harvested from P7 and P22 mice. The lens, cornea, and optic nerve were dissected and RNA was isolated using the RNeasy Mini Kit (Qiagen) according to the manufacturer’s protocol. RNA was additionally isolated from brain and muscle to serve as a positive and negative experimental control. cDNA was generated using the SuperScript II system (Thermo Fisher Scientific) following the recommended protocol. Samples processed without the addition of reverse transcriptase were used as negative controls in all of the above experiments. Quantitative RT-PCR was performed on the Applied Biosystems 7500 Real Time PCR System using the Taqman Assay system with inventoried probes for *Myrf, Tmem98, Mfrp, and Best1* (Thermo Fisher Scientific). Critical cycle threshold levels of each sample were normalized to levels of *Hprt*. Fold activity was calculated using the ddCt method [100] as above and reported relative to wild-type littermates.

### Animal use and genotyping

*Rxcre* mice [57] and *Myrf* conditional knockout mice [30] have been previously described. DNA was isolated from tail biopsies and was genotyped as described in **S5 Table**. Animals were also genotyped for *rd1, rd8, and rd10* according to established methods, to rule out co-segregation of common retinal degenerations in C57BL/6J mouse strains [101].

### Histology and immunofluorescence staining

Eyes or embryonic heads were fixed in 4% paraformaldehyde (PFA) 0.1 M NaPO_4_ pH 7.3 for 30–60 min at 22°C, dehydrated through a series of ethanols to 70% and then embedded in the Miles Scientific Tissue-Tek VP Model #20 embedding machine (Newark, DE, USA) and the Shandon Histocentre 2 Model #64000012 embedding station (Thermo Fisher Scientific) and sectioned at 6μm. Hematoxylin and eosin staining on paraffin sections was done according to established methods [102]. For immunostaining, paraffin was removed with xylene and slides were rehydrated and washed in phosphate buffered saline (PBS). Sections were incubated in phosphate buffered 3% H_2_O_2_ overnight at 22°C to quench autofluorescence of the RPE. Sections were boiled in 10mM Citric Acid, pH 6.0 for 7 min for antigen retrieval and allowed to recover for 30 minutes. Sections were blocked in 10% Normal Donkey Serum (NDS), 1% Bovine Serum Albumin (BSA) in PBTx (0.1 M NaPO_4_ pH 7.3 0.5% Triton X-100) for 2 hrs. The Mouse On Mouse Kit (Vector Labs, Burlingame, CA, USA) was used to reduce the background from antibodies generated in mouse. Sections were incubated in the following primary antibodies overnight at 4°C rabbit anti-TMEM98 (1:500, 14731-1-AP, Proteintech, Chicago, IL, USA), mouse anti-Rho (1:400, ab98887, Abcam, Cambridge, UK), rabbit anti-mCar (1:1000, AB15282, Millipore Sigma, Burlington, MA, USA), rabbit anti-GFP (1:100, ab6556, Abcam), rabbit anti-MFRP (1:500, af3445, R&D Biosystems, Minneapolis, MN, USA). After incubation in the primary antibody, sections were washed in PBS and incubated in a species-specific Alexafluor conjugated fluorescent secondary antibodies for two hours at 22°C. Sections were then rinsed in PBS, the nuclei were stained with 4′,6-diamidino-2-phenylindole (DAPI) and coverslips were adhered to slides with ProLong Gold Antifade mounting media (Thermo Fisher Scientific). Slides were imaged using the Leica SP5 confocal system (Leica, Wetzlar, Germany) or an Olympus BX-51 epifluorescence microscope (Olympus, Center Valley, PA, USA).

### Retinal layer measurements, whole eye measurements, and cell counting

Eyes from P10, P14, and P22 animals were enucleated and aligned such that the optic nerve was inline with the central cornea and both could be readily observed, and images were captured on the Leica MX10F dissecting microscope (Leica, Wetzlar, Germany). Ocular axial length for the P22 time point was measured using ImageJ 1.51m9 software [103], by marking a line from the central cornea to the base of the optic nerve. For the analysis, the following number of animals/eyes were used: P22 control, n = 7 animals, 14 eyes; P22 *Rxcre;Myrf*^*+/fl*^ n = 3 animals, 6 eyes; *Rxcre;Myrf*^*fl/fl*^, n = 3 animals, 6 eyes. Additional eyes at each time point were visually inspected but not systematically imaged or measured. Images from P22 sections stained with mCar, Rho, and DAPI were used to determine the thickness and area of retinal layers, and the fraction of rods and cones within the retina in ImageJ (overlapping with some of the axial length measurement cohorts above) [103]. A minimum of 4 animals per genotype and 3 sections per animal were used for calculations (**Fig 6, S4 Table**). The percentage of each layer was determined by measuring the area of a particular layer and comparing it to the total retinal area excluding the inner/outer segments as the area of these was disproportionately reduced in the *Rxcre;Myrf*^*fl/fl*^ mice (**Fig 6**). Areas of the outer nuclear layer (ONL), inner nuclear layer (INL), and ganglion cell layer (GCL) were determined by measuring the area of each layer in DAPI stained sections. The total areas of the outer segment and inner segment were determined by measuring these layers in Rho/DAPI stained sections. The total number of nuclei in the ONL was determined by sampling 3 comparable sized regions of each low power DAPI image, averaging the three counts to obtain the cell density and multiplying that number by the total area of the ONL. The percentage of cones as a fraction of total retinal cells was determined by calculating the number of cones in the outer nuclear layer, stained by mouse cone-arrestin (mCar), and comparing it to the total number of DAPI stained cells in the ONL, INL, and GCL. Cells in the INL and GCL were counted directly. The Student’s t-test, two tailed, unequal variance was used to determine statistical significance.

### RPE flat mounts

Eyes were enucleated from P7 and P22 mice, and cornea, lens, optic nerve, and retina tissues were removed. At least 3 animals/eyes per genotype per time point were used. P7 eyes were treated for 5 minutes with PBTx to aid in the separation of the retina from the RPE. RPE was fixed for 45 minutes in phosphate buffered 4% paraformaldehyde. After fixation, RPE samples were rinsed in PBS and incubated in phosphate buffered 3% H_2_O_2_ for 48 hours at 4°C to quench the RPE autofluorescence. RPE samples were washed in PBS and blocked in 10% Normal Goat Serum (NGS), 1% BSA in PBTx for 2 hours. Primary antibody incubation occurred overnight at 4°C with the antibodies and dilutions to the following antigens: TMEM98 (1:500, 14731-1-AP, Proteintech), MFRP (1:500, af3445, R&D Biosystems, Minneapolis, MN, USA). RPE samples were washed in PBS and incubated with Alexa-conjugated fluorescent secondary antibodies for 2 hours at 22°C. RPE samples were rinsed in PBS and the nuclei were stained with DAPI. A series of 3–4 radial cuts were made to flatten the RPE and underlying scleral and samples were mounted with Prolong Gold Antifade. Images were taken on a Leica SP5 confocal microscope or Olympus BX-51 epifluorescence microscope.

### Mouse ocular imaging and electrophysiology

Eyes from 10-month old control (*Myrf* ^*fl/fl*^ or *Myrf* ^*+/fl*^; n = 4 eyes, 2 mice), heterozygous (*RxCre;Myrf* ^*+/fl*^; n = 4 eyes, 2 mice), and homozygous knockout mice (*RxCre;Myrf* ^*fl/fl*^; n = 12 eyes, 6 mice) from a single cohort (separate from the histology cohort) were evaluated *in vivo* sequentially by fundus photography, spectral domain OCT, and electroretinography. Mice were anesthetized with an intraperitoneal (IP) injection of ketamine (50 mg/kg) and xylazine (5 mg/kg). The eyes were dilated with drops of 1% tropicamide and 2.5% phenylephrine. Central and peripheral linear scans were obtained on each mouse eye using an Envisu 2200 spectral domain ophthalmic imaging system (SD-OCT, Bioptigen, Morrisville, NC). Rectangular volume scans consisting of 1000 A-scans by 100 B-scans over a 1.4 x 1.4 mm area centered on the optic nerve head and the peripheral section were also taken for visualization of retinal anatomy. Similar rectangular volume scans centered on the anterior chamber and vitreous chamber were acquired sequentially on a separate cohort of P22 mice (control: n = 8 eyes, 4 mice; *RxCre;Myrf* ^*+/fl*^: n = 6 eyes, 3 mice; *RxCre;Myrf* ^*fl/fl*^: n = 12 eyes, 6 mice). Anterior chamber depth was measured from the posterior surface of the central cornea to the anterior surface of the lens using clippers in the Bioptogen software. Due to image quality, anterior chamber depth could not be measured on 1 *RxCre;Myrf* ^*+/fl*^ and 1 *RxCre;Myrf* ^*fl/fl*^ eye. Vitreous chamber depth was measured as the average distance between the posterior lens capsule and the surface of the retinal nerve fiber layer measured at four points at a distance 350 μm from the center of the optic nerve head, using the Bioptogen Diver software; retinal thickness was measured similarly on these images from the edge of the nerve fiber layer to the reflecting band of the RPE (**S6D Fig**). For 2 eyes from one *RxCre;Myrf* ^*+/fl*^ mouse, the vitreous chamber depth and retinal thickness could only be measured at 3 points due to image quality. Statistical comparisons between *RxCre;Myrf* ^*+/fl*^ or *RxCre;Myrf* ^*fl/fl*^ eyes and controls were done with one-way ANOVA in Graphpad Prism 7 (San Diego, CA, USA), and subsequent pairwise comparison were done with two-tailed Student’s *t*-test in cases of significance. Central retinal fundus photography was performed using the Micron III retinal imaging system (Phoenix Research Labs, Pleasanton, CA).

For electroretinography, the 10-month old mice described above were anesthetized and dilated as above. An additional drop of 0.5% proparacaine was used to anesthetize the eyes. An electrode and stimulator were placed onto the surface of the eye and cushioned with a drop of 0.3% hypromellose (GenTeal, Novartis, Landsville, PA). A Diagnosys Celeris System Electrophysiology System (Diagnosys, Lowell, MA) was used to measure responses to stimuli. Mice were dark adapted overnight and scotopic responses were recorded sequentially using 0.01, 10, and 32 cd*s/m^2^ intensity stimuli. Mice were then light adapted for 10 minutes prior to photopic testing. Photopic responses were recorded sequentially using 10, 32, and 100 cd*s/m^2^ stimuli. Photopic flicker responses were then recorded using 20 cd*s/m^2^ cycled at 9.9 Hz to suppress rod responses. During testing, body temperature was maintained at 37°C using the built-in heater. Statistical comparison of a-wave and B-wave amplitudes between *RxCre;Myrf* ^*fl/fl*^ or *RxCre;Myrf* ^*+/fl*^ eyes and control littermates was done using two-tailed Student’s *t*-test in Microsoft Excel. Given that no significant a-waves were observed in the scotopic dim flash (0.1 cd*s/m^2^) or any of the photopic flashes for our control mice, we did not compare a-wave amplitudes for these stimuli.

### Western blot analysis and co-immunoprecipitation

Tagged MYRF and TMEM98 expression constructs were transfected into HEK-293T cells using Lipofectamine 2000 (Thermo Fisher Scientific) as per manufacturer’s instructions, with 1.5μg of total DNA used per well in a 6-well plate. Forty-eight hours after transfection, cells were lysed in cold RIPA buffer (150mM NaCl, 1% Nonidet P-40, 0.5% sodium deoxycholate, 0.1% SDS and 25mM Tris pH7.4) supplemented with EDTA-free protease inhibitor tablets (Roche). Lysates were clarified at 13,000 RPM for 15 minutes at 4°C, diluted in Laemmli buffer (Sigma), denatured at 95°C for 5 minutes, run on 4–12% Bis-tris gradient gels (Thermo Fisher Scientific), and transferred to PVDF membrane (Thermo Fisher Scientific). Blots were blocked in 5% skim milk powder in Tris-buffered saline with 0.1% tween-20 (TBST) for 1 hour and probed with primary antibodies overnight at 4°C diluted in 1% BSA in TBST. The primary antibodies and dilutions used were: mouse anti-Myc (4A6, Millipore) at 1:1,000; rat anti-HA (3F10, Roche) at 1:1,000, mouse anti-β-Tubulin at 1:100 (Developmental Studies Hybridoma Bank), and rabbit anti-Flag at 1:1,000 (Cell Signaling, Danvers, MA, USA). Washed blots were then probed for 1 hour with appropriate HRP-linked secondary antibodies (1:5,000, Cell Signaling) and imaged using the Supersignal West Pico Chemiluminescent substrate (Thermo Fisher Scientific) on the Syngene G:Box iChemi XT Gel Imaging system (Syngene, Frederick, MD, USA).

For co-immunoprecipitation experiments, transfected cells were lysed in cell lysis buffer (20mM Tris pH7.5, 150mM NaCl, 1% Triton X-100, 1mM EDTA, 1mM EGTA) supplemented with EDTA-free protease inhibitor tablets (Roche), and clarified at 13,000 RPM for 15 minutes at 4°C. Five percent of the clarified supernatants was kept for inputs. The remainder was incubated for 1 hour with 1 μg of rat anti-HA (3F10, Roche) or mouse anti-Myc (4A6, Millipore) at 4°C with agitation and subsequently for 2 hours with 40μl of Dynabeads Protein G (Thermo Fisher Scientific). Beads were then washed three times with cell lysis buffer and bound antibody and protein eluted in 2x Laemmli buffer at 95°C for 5 minutes. Eluted protein samples were run on 4–12% Bis-tris gradient gels and probed as above using 1:1,000 4A6 mouse anti-Myc (Millipore) or 1:2,000 HRP-conjugated rabbit anti-HA (Cell Signaling).

## Supporting information

S1 Fig Exclusion of *BEST1* as the underlying cause for NNO1. (A) Schematic of *BEST1* gene, showing screening of primer sets used in addition to sequencing all of the coding exons. (B) Supplemental screening primer sequences.
(TIF)

S2 Fig Pooled exome haplotype enrichment strategy. (A) Schematic description of pooled approach. By pooling all of the affected individuals and using a matched unaffected pool of their family members, we enrich for sequence reads from the disease haplotype (red). (B) *In silico* analysis of predicted difference in allele frequency (ΔAF) from each haplotype based on the pooling strategy. The disease haplotype (red) is expected to be represented in 50% of the reads (0.5 ΔAF), while variants on the other haplotypes should be present in less than 5% of reads (0.05 ΔAF).
(TIF)

S3 Fig Evolutionary constraint of top candidate genes *MYRF* and *PPP1R32* based on gnomAD data. *MYRF*, in contrast to *TMEM98*, is constrained against loss of function loss of function variants, with only 2 observed in the gnomAD cohort; it is also moderately constrained against missense variation. In contrast, *PPP1R32* does not show significant evolutionary constraint to loss of function or missense variants.
(TIF)

S4 Fig Confirmation of segregation of *ZP1* and *PPP1R32* variants in the NNO1 family. Agarose gel electrophoresis for *Sbf*I (top) or *Pvu*I (bottom) restriction digest of PCR products for *ZP1* and *PPP1R32*, respectively, used to confirm variant segregation in NNO1 family within one large nuclear family branch.
(TIF)

S5 Fig MYRF antibody staining in the retina and RPE. (A-C) MYRF staining in P22 (A), P3 (B) mouse eyes or adult human eye (C) using the validated N-terminal MYRF antibody. Left panels show MYRF antibody staining (green) and counterstaining of nuclei with DAPI. Right side shows controls stained under the same conditions with no primary antibody. There is signal in retinal pigment epithelial cells and nonspecific signal in the retina.
(TIF)

S6 Fig Loss of MYRF does not grossly affect eye size in mice. (A-C) Whole eye photographs from control, *RxCre;Myrf*^*+/fl*^, *RxCre;Myrf*^*fl/fl*^ and from P10 (A), P14 (B) or P22 (C) mice. (D) Representative images of posterior segment SD-OCT for control, *RxCre;Myrf*^*+/fl*^, *RxCre;Myrf*^*fl/fl*^ eyes used for measuring retinal thickness and vitreous chamber. Red line indicates location for retinal thickness measurements and blue line indicates location for vitreous chamber depth (VCD) measurements. (E) Quantitative analysis of axial length measurements from P22 enucleated eyes. There is no statistically significant difference in eye size across using pairwise comparisons across each pair of genotypes (two tailed Student’s *t*-test) for this time point. (F-H) Quantitative analysis of retinal thickness (F), VCD (G), and anterior chamber depth (ACD) based on SD-OCT measurements from P22 eyes. There is a small, but significant dose dependent decrease in retinal thickness in conditional knockout mice by one-way ANOVA and subsequent pairwise *t*-test comparisons. Otherwise, there is no biologically meaningful difference in the ACD and VCD parameters among the genotypic groups. Scale bar, 1 mm in A-C; 200 μm in D. ***, p<0.001, ** p<0.01, * p<0.05.
(TIF)

S7 Fig Early RPE pigmentation is preserved in Myrf CKO mice. Sections from E13.5 wild-type, *RxCre;Myrf*^*+/fl*^, *RxCre;Myrf*^*fl/fl*^ eyes showing preservation of RPE pigmentation and no appreciable difference between genotypes. Discontinuity in pigmentation corresponds to the area of optic nerve. Scale bar, 250 μm.
(TIF)

S8 Fig Lineage tracing of *RxCre* with *RosafloxYFP* reporter. There is uniform YFP staining in the retina and the RPE in wild-type *RxCre;RosafloxYFP* mice and *RxCre;Myrf*^*fl/fl*^*;RosafloxYFP* mice. Scale bar, 250 μm; inset scale bar, 100 μm.
(TIF)

S9 Fig Histologic analysis of *Myrf*^*+/-*^ and *RxCre;Myrf*^*fl/-*^ mice. Hematoxylin and eosin staining of P22 adult sections from these mice shows no appreciable RPE or retinal phenotype *Myrf*^*+/-*^ mice, and decreased RPE pigmentation with photoreceptor loss and outer segment shortening in *RxCre;Myrf*^*fl/-*^ mice.
(TIF)

S10 Fig TMEM98 expression in developing and adult retinal sections. TMEM98 expression is confined largely to the RPE, with weaker expression in retina and sclera in P22 mice. The level of expression is much weaker in *RxCre; Myrf*^*fl/fl*^, similar to that observed in flatmounts.
(TIF)

S11 Fig MFRP expression in adult retinal section. MFRP expression is confined to the RPE and there is no appreciable difference in expression pattern or level among the genotypes.
(TIF)

S1 Table Pooled exome candidate variants for the NNO1 linkage region. (PDF)

S2 Table *MYRF* variants in nanophthalmos/high hyperopia probands. (PDF)

S3 Table *MYRF* variants in selected individuals from The Genomic Ascertainment Cohort (TGAC). (PDF)

S4 Table Cell count data from *Myrf* conditional knockout mice. (PDF)

S5 Table Electrophysiology data on *Myrf* conditional knockout mice. (PDF)

S6 Table MYRF screening primers and conditions. (PDF)

S7 Table Other primers and PCR conditions used in this study. (PDF)

S1 Data Primary data for qRT-PCR experiment in Fig 5A. (XLSX)

S2 Data Primary data for qRT-PCR experiment in Fig 8A and 8B. (XLSX)
