# Supplementary figures and images for: Variants in myelin regulatory factor (MYRF) cause autosomal dominant and syndromic nanophthalmos in humans and retinal degeneration in mice
Source: PLoS Genet. 2019 May 2;15(5):e1008130. doi: 10.1371/journal.pgen.1008130 (PMC6527243; doi:10.1371/journal.pgen.1008130)

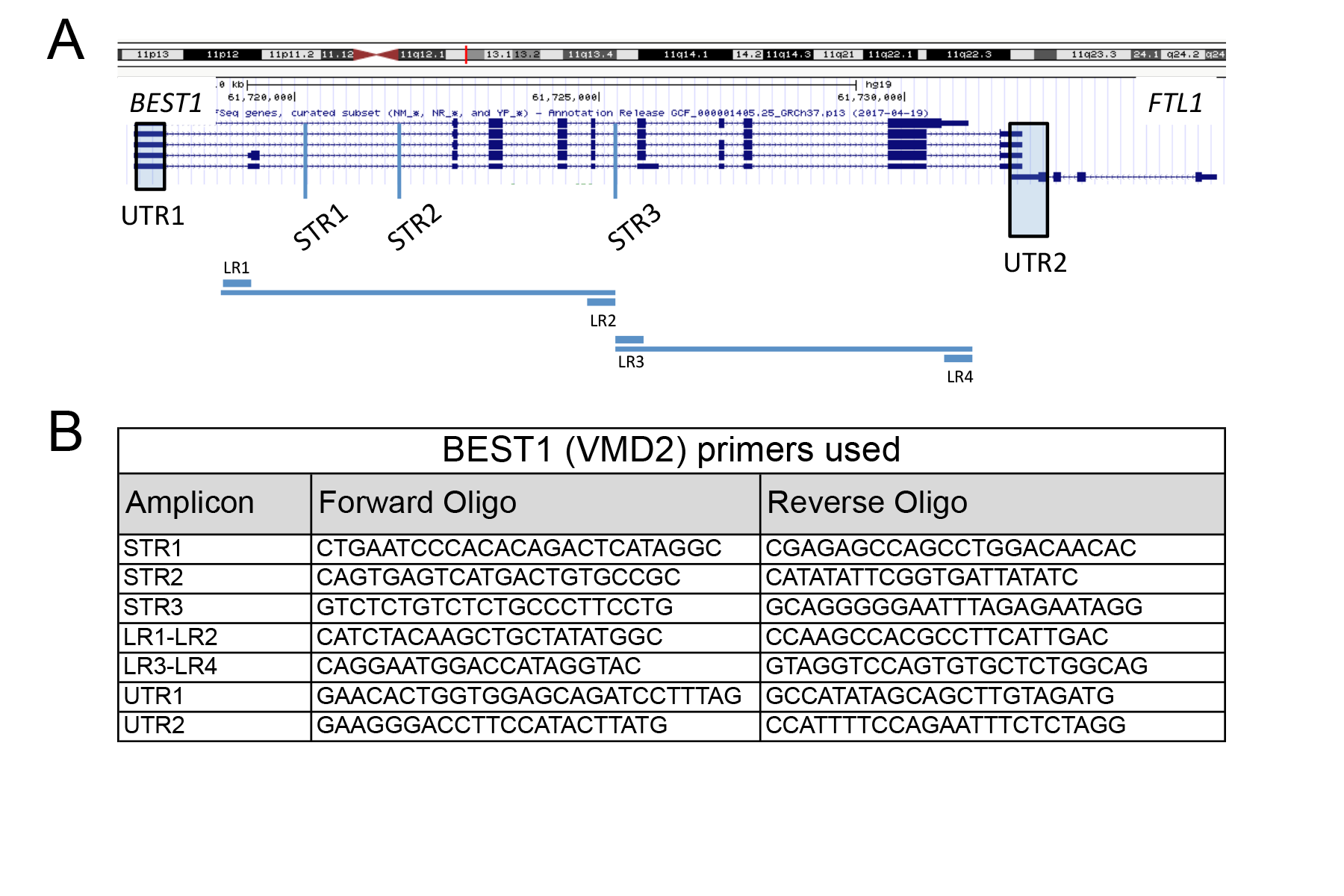

Supplement: S1 Fig — (A) Schematic of BEST1 gene, showing screening of primer sets used in addition to sequencing all of the coding exons. (B) Supplemental screening primer sequences. (TIF) [file pgen.1008130.s001.tif]

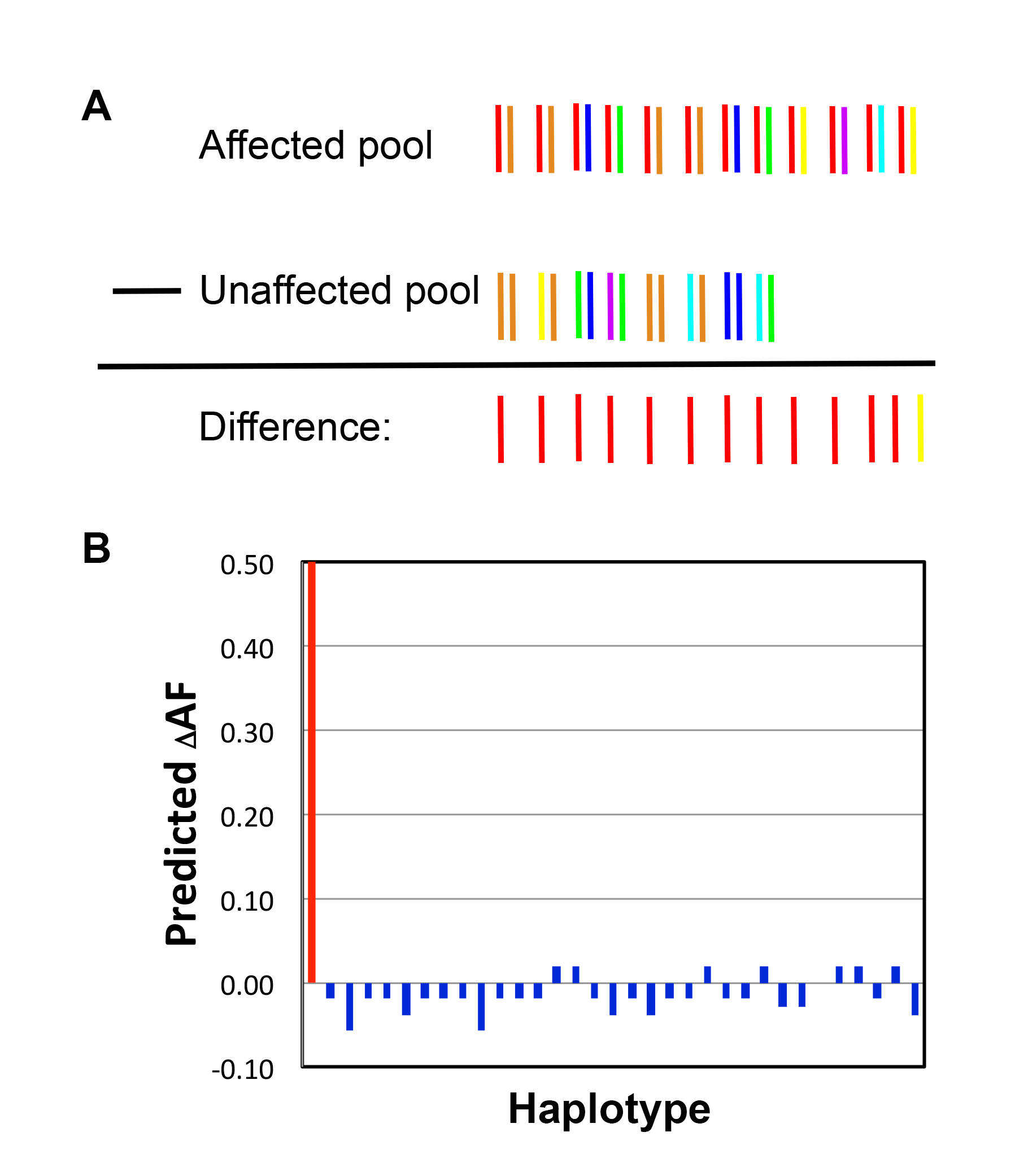

Supplement: S2 Fig — (A) Schematic description of pooled approach. By pooling all of the affected individuals and using a matched unaffected pool of their family members, we enrich for sequence reads from the disease haplotype (red). (B) In silico analysis of predicted difference in allele frequency (ΔAF) from each haplotype based on the pooling strategy. The disease haplotype (red) is expected to be represented in 50% of the reads (0.5 ΔAF), while variants on the other haplotypes should be present in less than 5% of reads (0.05 ΔAF). (TIF) [file pgen.1008130.s002.tif]

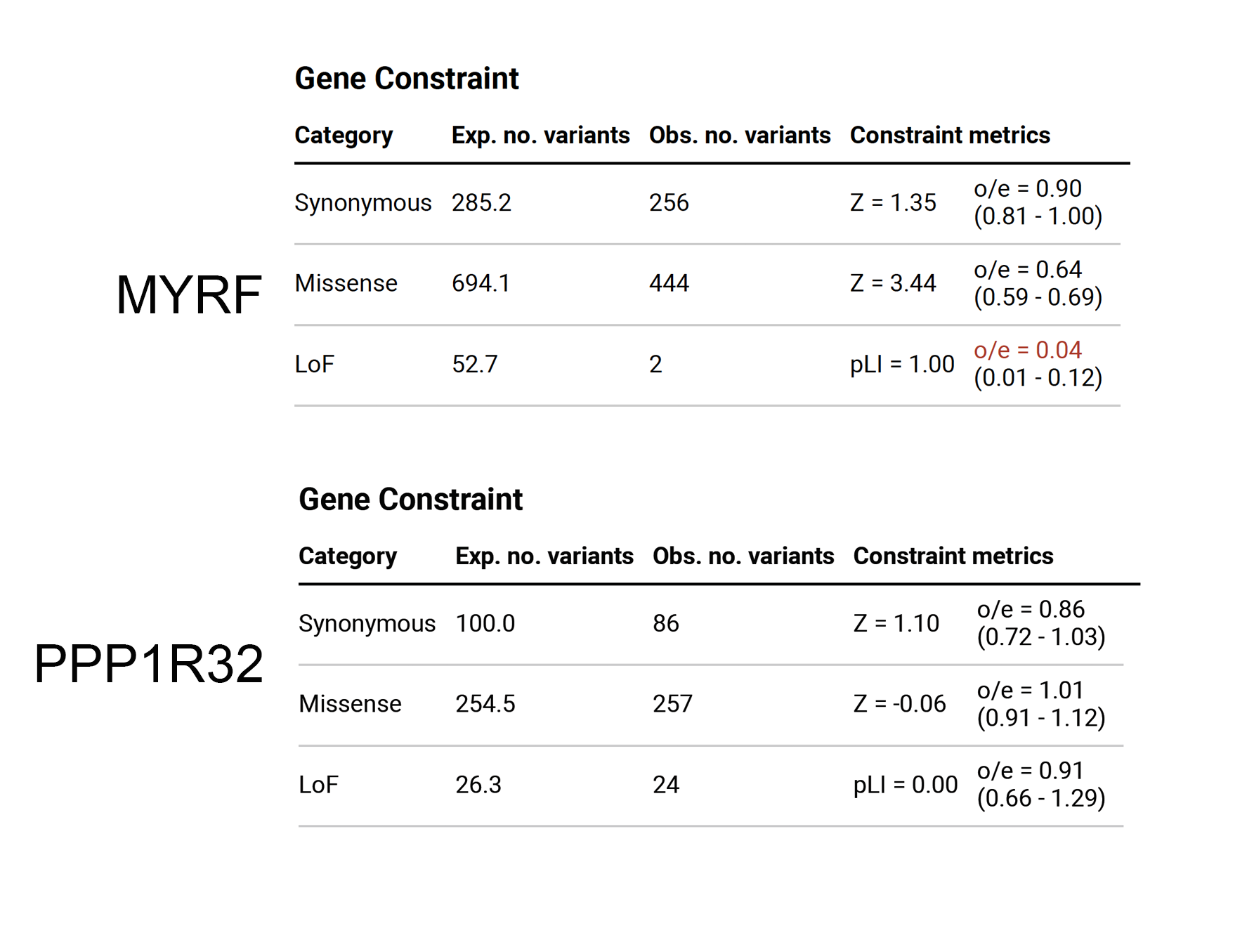

Supplement: S3 Fig — MYRF, in contrast to TMEM98, is constrained against loss of function loss of function variants, with only 2 observed in the gnomAD cohort; it is also moderately constrained against missense variation. In contrast, PPP1R32 does not show significant evolutionary constraint to loss of function or missense variants. (TIF) [file pgen.1008130.s003.tif]

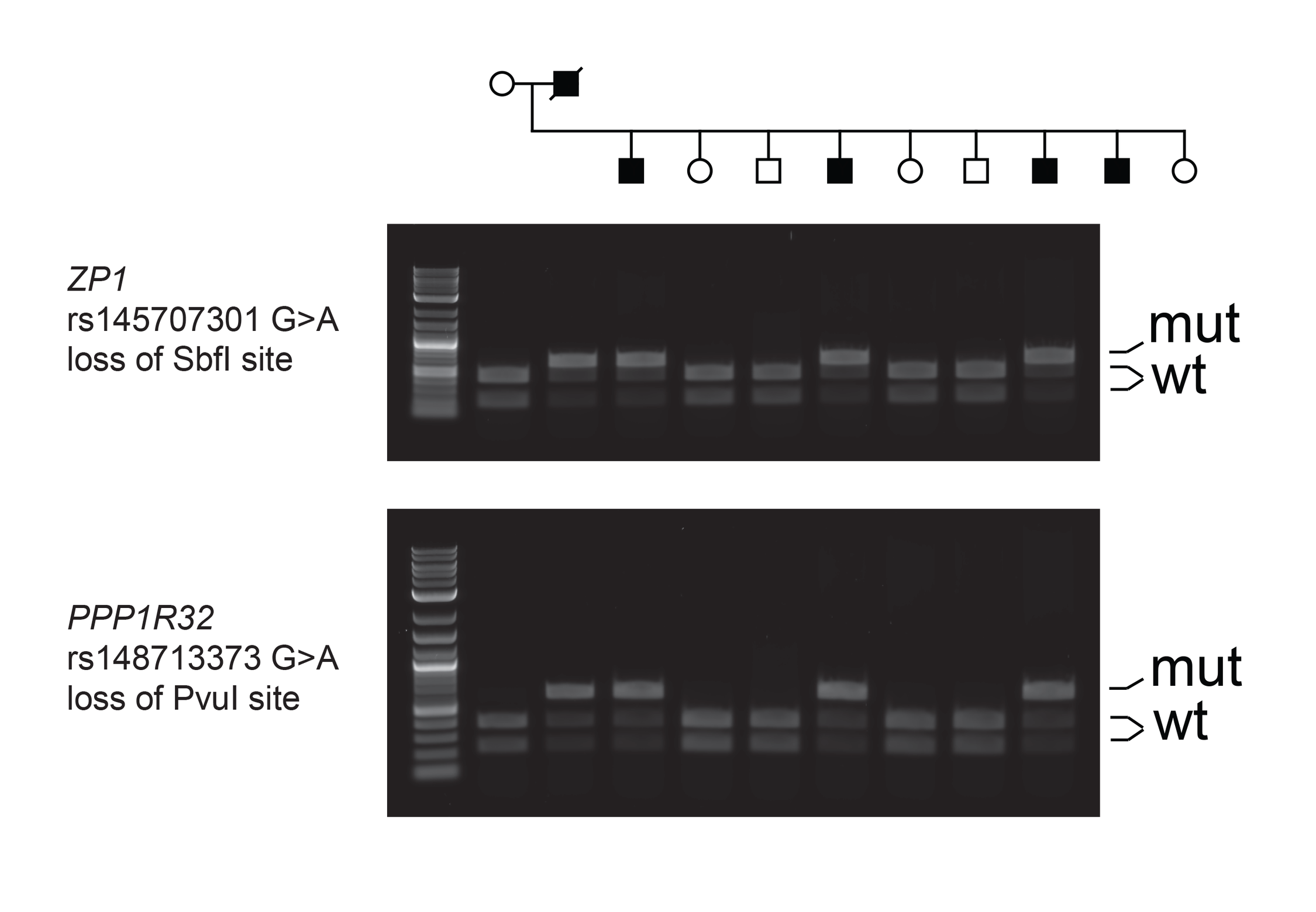

Supplement: S4 Fig — Agarose gel electrophoresis for SbfI (top) or PvuI (bottom) restriction digest of PCR products for ZP1 and PPP1R32, respectively, used to confirm variant segregation in NNO1 family within one large nuclear family branch. (TIF) [file pgen.1008130.s004.tif]

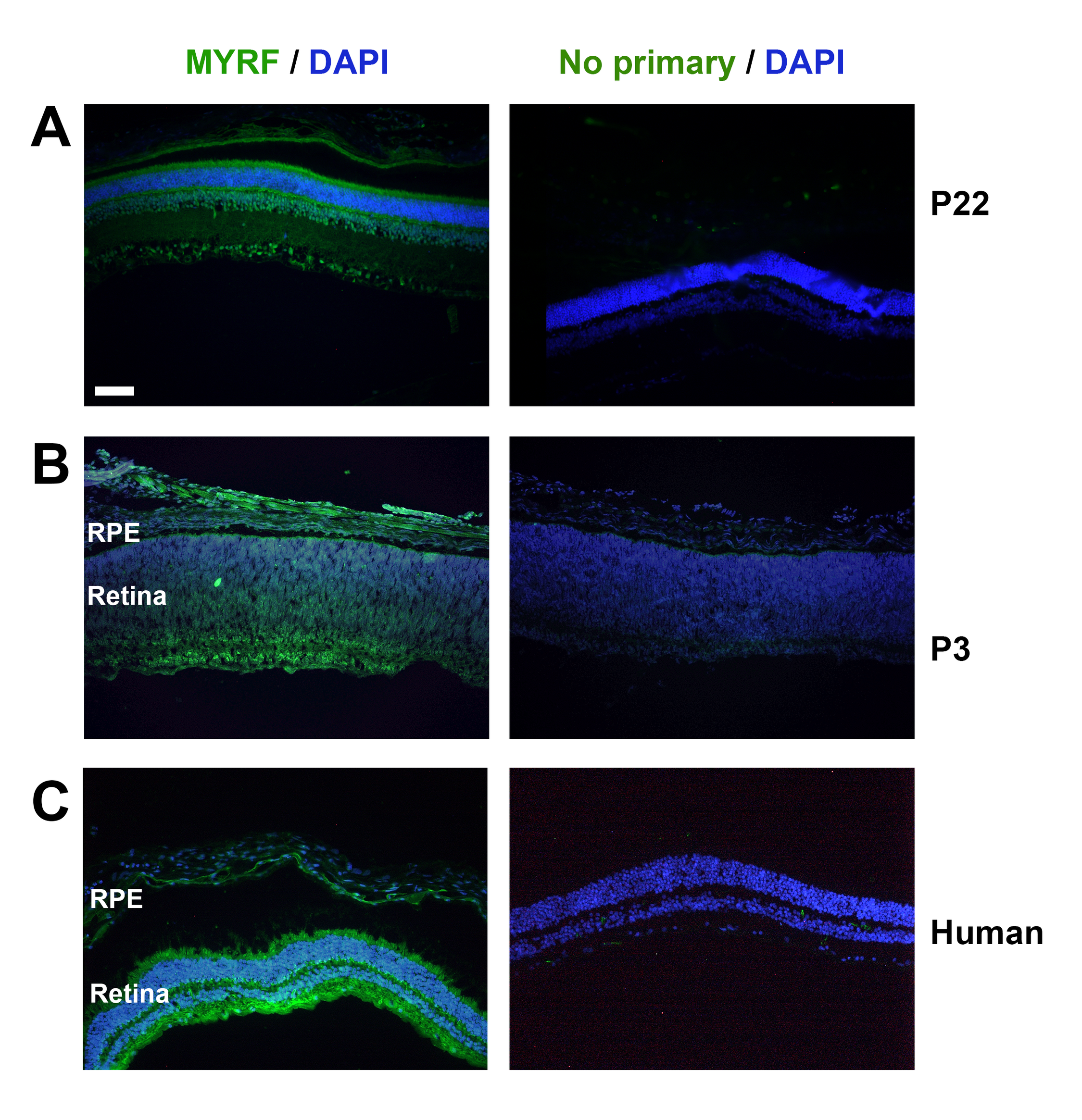

Supplement: S5 Fig — (A-C) MYRF staining in P22 (A), P3 (B) mouse eyes or adult human eye (C) using the validated N-terminal MYRF antibody. Left panels show MYRF antibody staining (green) and counterstaining of nuclei with DAPI. Right side shows controls stained under the same conditions with no primary antibody. There is signal in retinal pigment epithelial cells and nonspecific signal in the retina. (TIF) [file pgen.1008130.s005.tif]

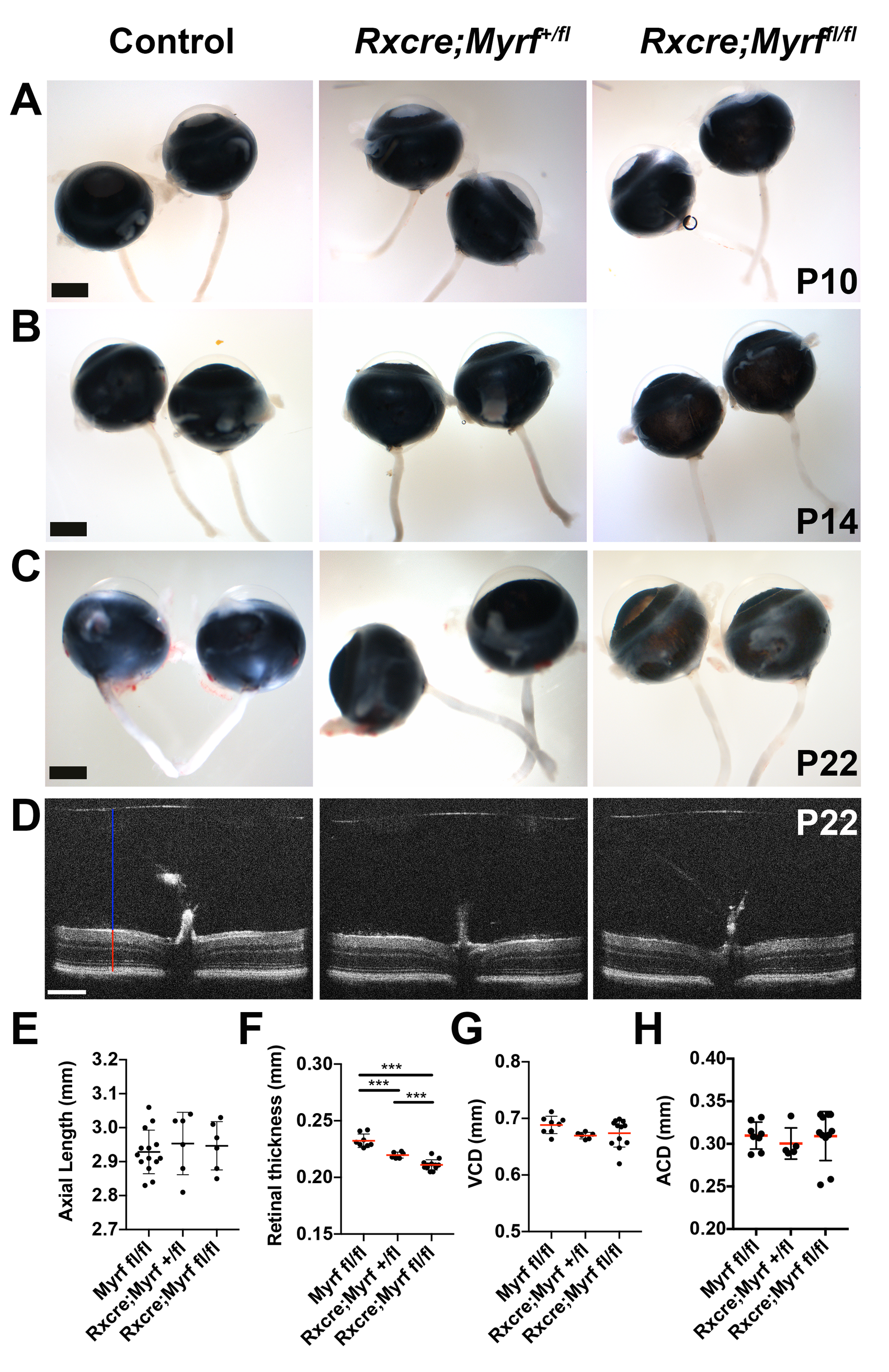

Supplement: S6 Fig — (A-C) Whole eye photographs from control, RxCre;Myrf+/fl, RxCre;Myrffl/fl and from P10 (A), P14 (B) or P22 (C) mice. (D) Representative images of posterior segment SD-OCT for control, RxCre;Myrf+/fl, RxCre;Myrffl/fl eyes used for measuring retinal thickness and vitreous chamber. Red line indicates location for retinal thickness measurements and blue line indicates location for vitreous chamber depth (VCD) measurements. (E) Quantitative analysis of axial length measurements from P22 enucleated eyes. There is no statistically significant difference in eye size across using pairwise comparisons across each pair of genotypes (two tailed Student’s t-test) for this time point. (F-H) Quantitative analysis of retinal thickness (F), VCD (G), and anterior chamber depth (ACD) based on SD-OCT measurements from P22 eyes. There is a small, but significant dose dependent decrease in retinal thickness in conditional knockout mice by one-way ANOVA and subsequent pairwise t-test comparisons. Otherwise, there is no biologically meaningful difference in the ACD and VCD parameters among the genotypic groups. Scale bar, 1 mm in A-C; 200 μm in D. ***, p<0.001, ** p<0.01, * p<0.05. (TIF) [file pgen.1008130.s006.tif]

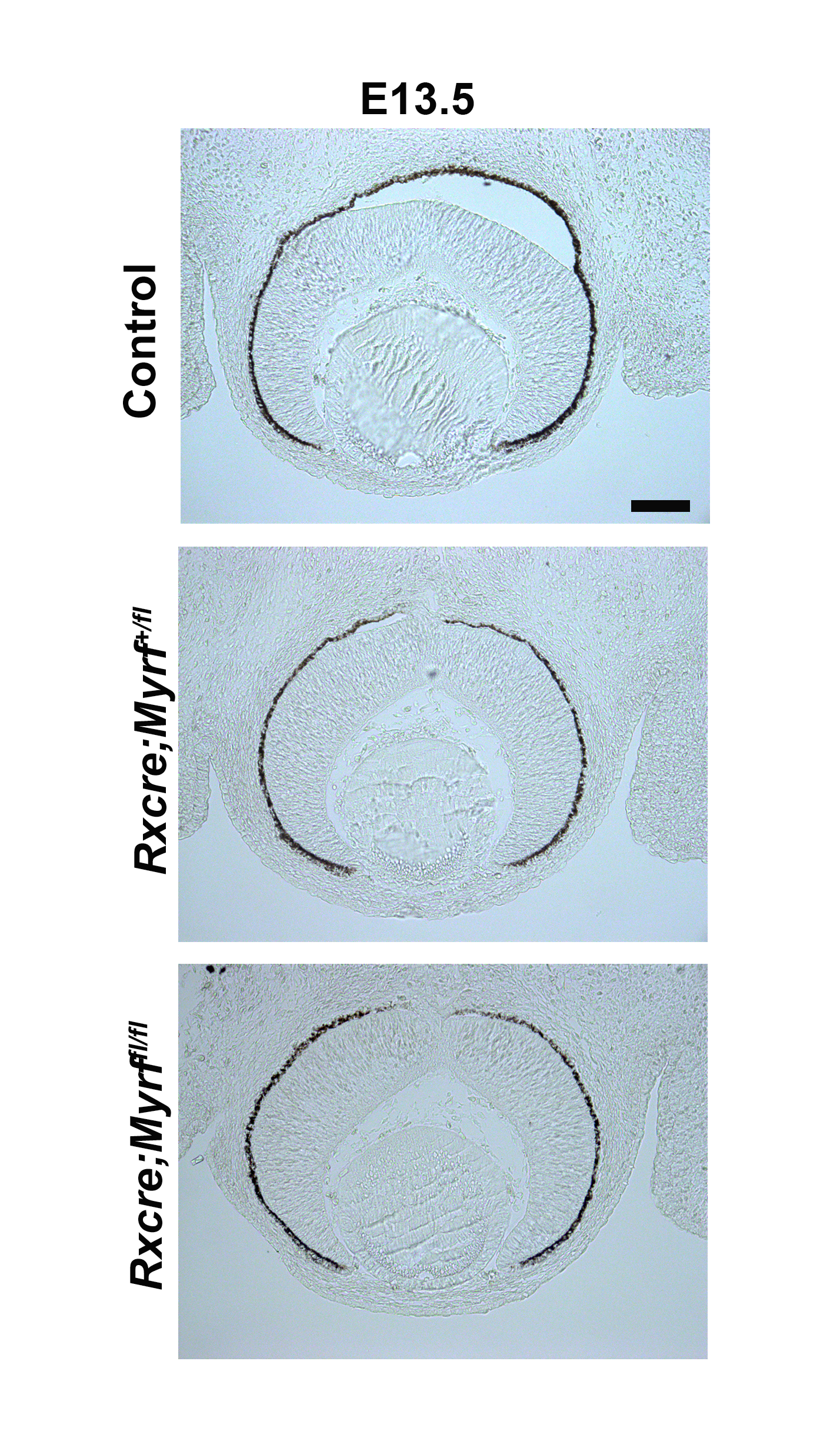

Supplement: S7 Fig — Sections from E13.5 wild-type, RxCre;Myrf+/fl, RxCre;Myrffl/fl eyes showing preservation of RPE pigmentation and no appreciable difference between genotypes. Discontinuity in pigmentation corresponds to the area of optic nerve. Scale bar, 250 μm. (TIF) [file pgen.1008130.s007.tif]

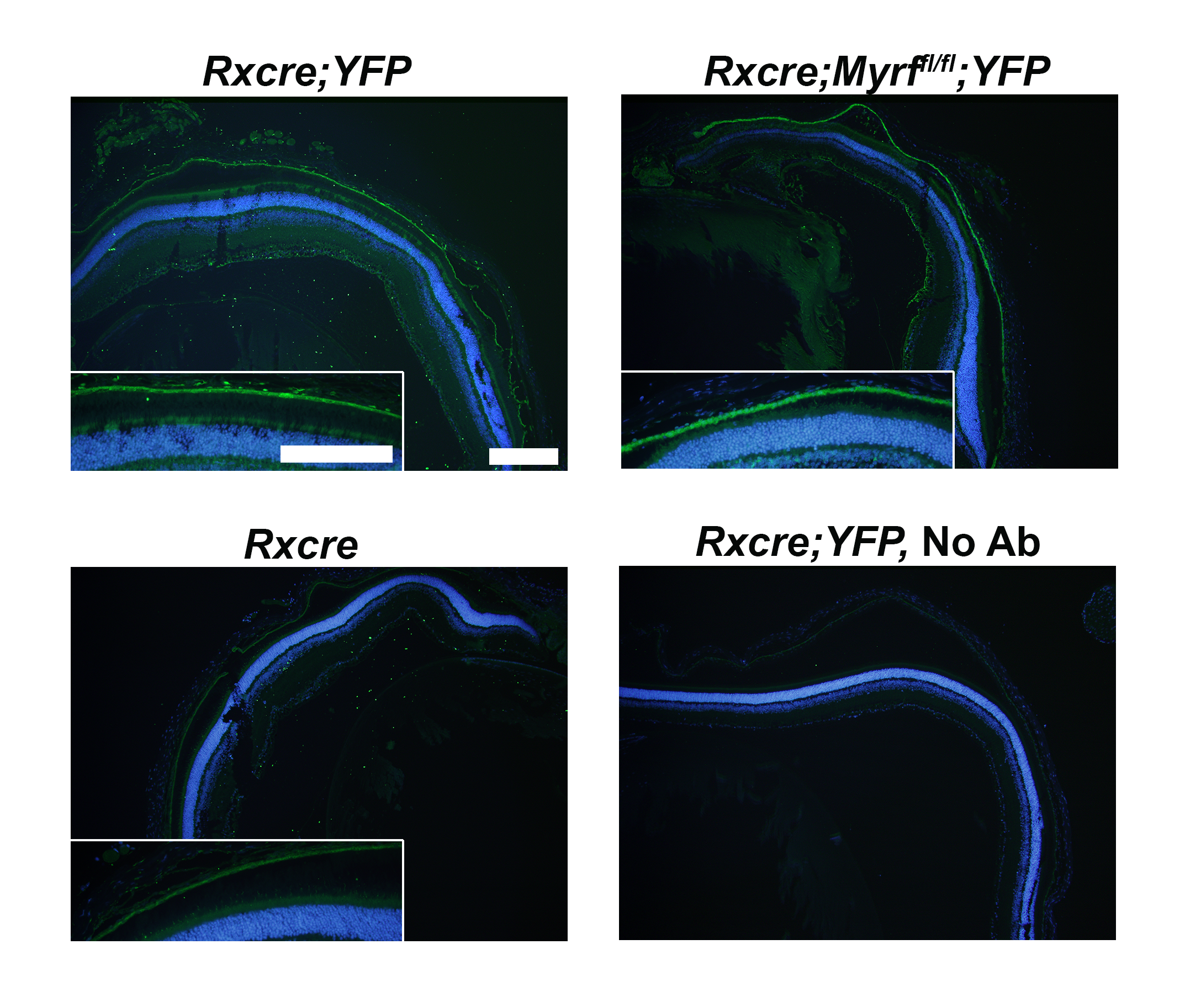

Supplement: S8 Fig — There is uniform YFP staining in the retina and the RPE in wild-type RxCre;RosafloxYFP mice and RxCre;Myrffl/fl;RosafloxYFP mice. Scale bar, 250 μm; inset scale bar, 100 μm. (TIF) [file pgen.1008130.s008.tif]

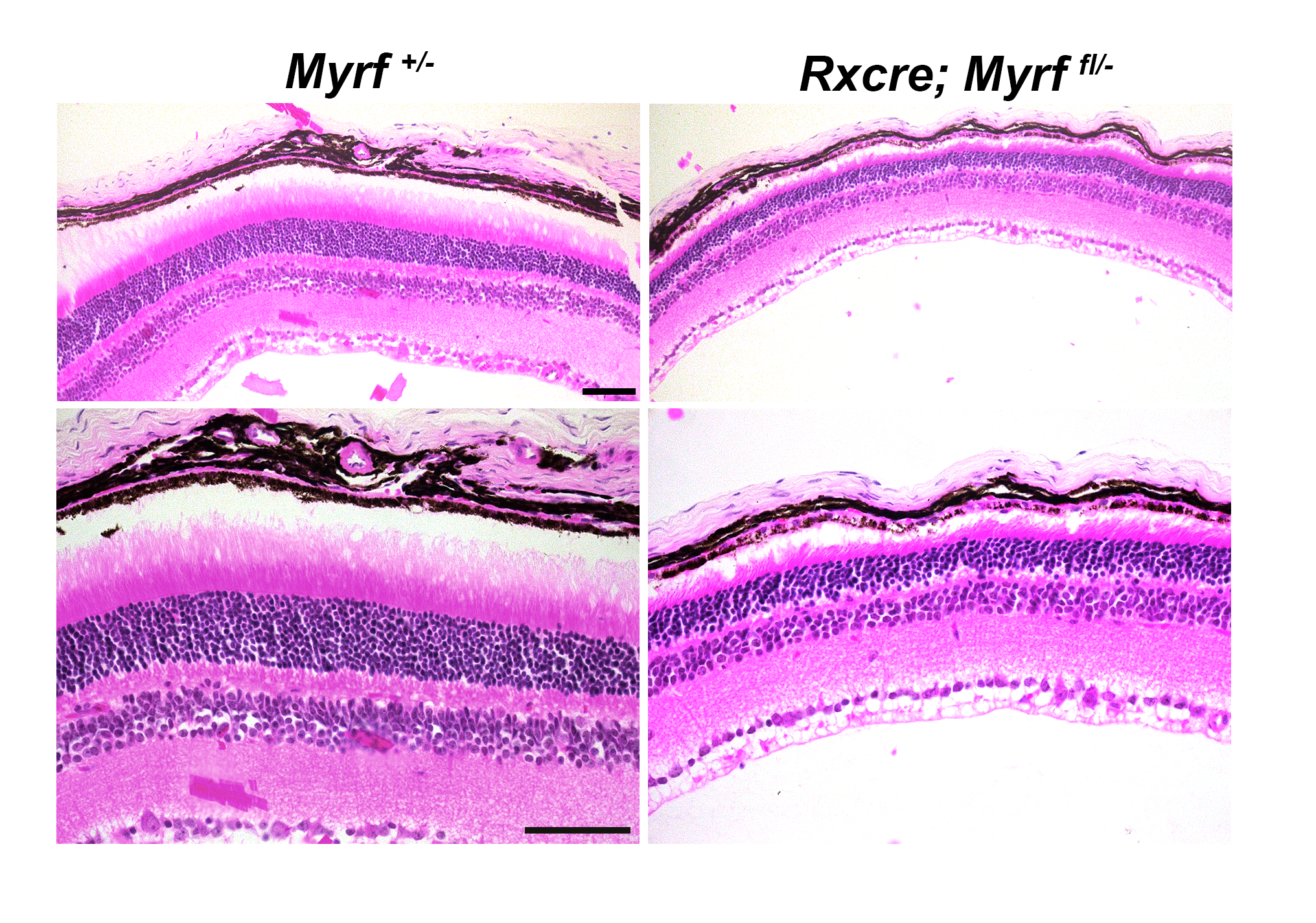

Supplement: S9 Fig — Hematoxylin and eosin staining of P22 adult sections from these mice shows no appreciable RPE or retinal phenotype Myrf+/- mice, and decreased RPE pigmentation with photoreceptor loss and outer segment shortening in RxCre;Myrffl/- mice. (TIF) [file pgen.1008130.s009.tif]

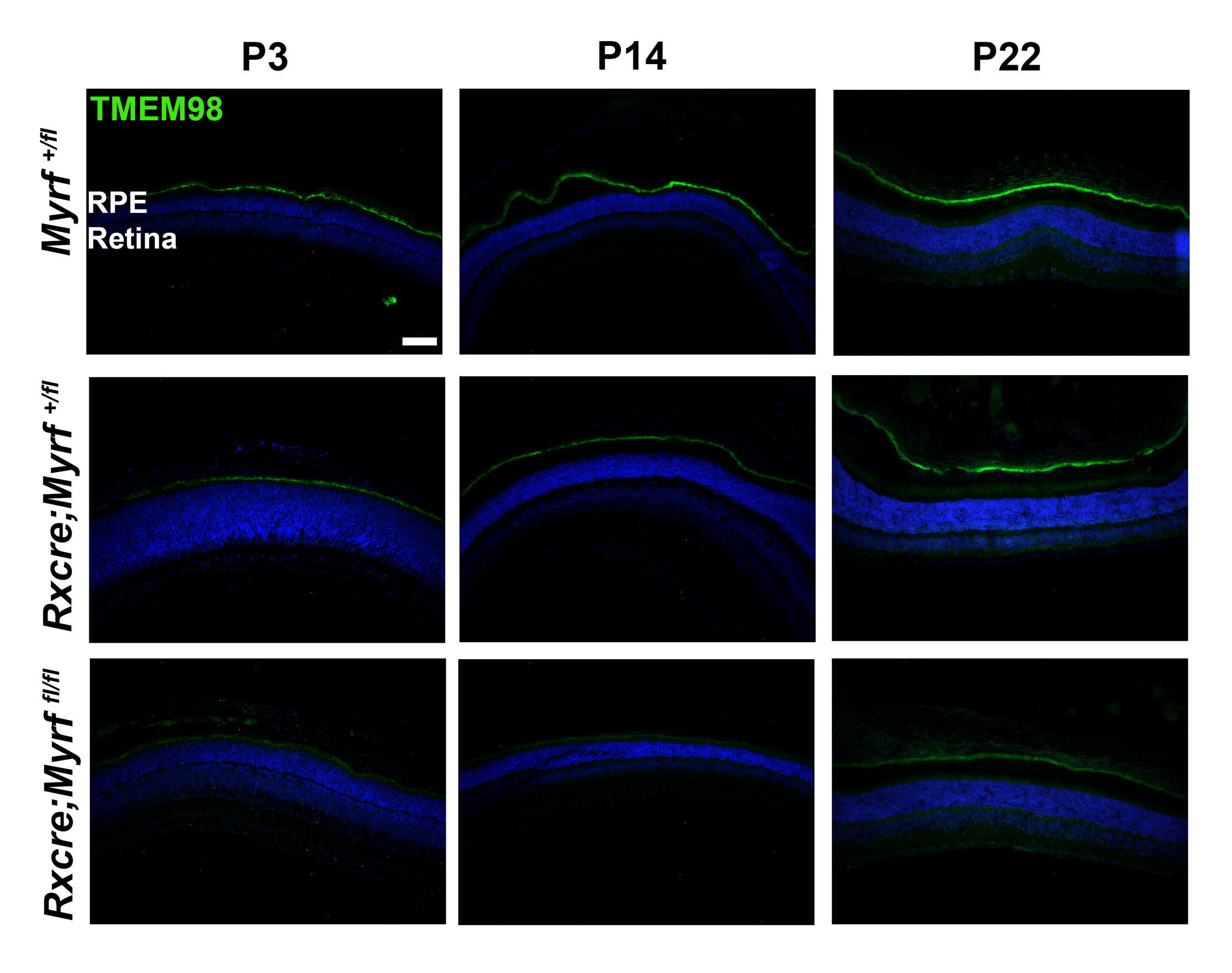

Supplement: S10 Fig — TMEM98 expression is confined largely to the RPE, with weaker expression in retina and sclera in P22 mice. The level of expression is much weaker in RxCre; Myrffl/fl, similar to that observed in flatmounts. (TIF) [file pgen.1008130.s010.tif]

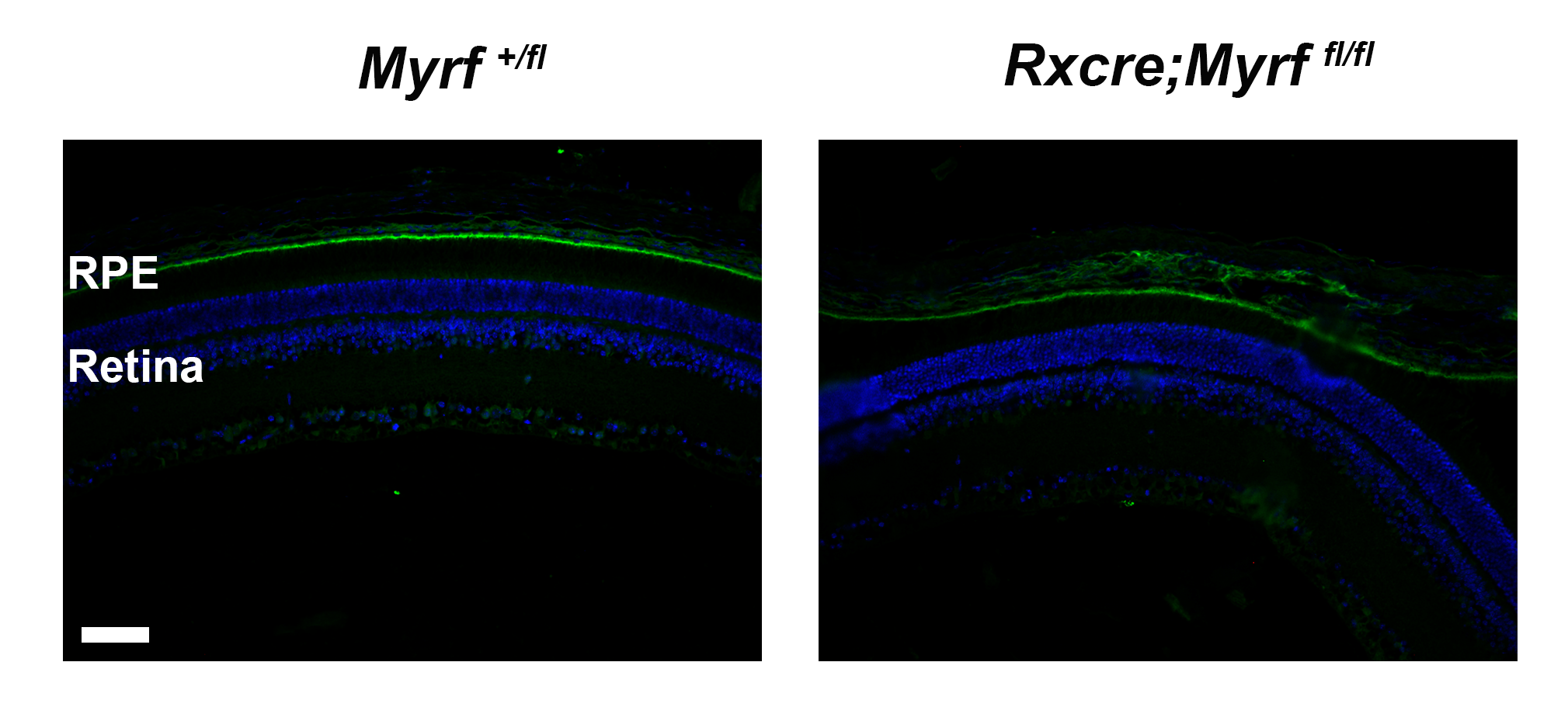

Supplement: S11 Fig — MFRP expression is confined to the RPE and there is no appreciable difference in expression pattern or level among the genotypes. (TIF) [file pgen.1008130.s011.tif]
