# Supplementary material for: Variants in myelin regulatory factor (MYRF) cause autosomal dominant and syndromic nanophthalmos in humans and retinal degeneration in mice
Source: PLoS Genet. 2019 May 2;15(5):e1008130. doi: 10.1371/journal.pgen.1008130 (PMC6527243; doi:10.1371/journal.pgen.1008130)
Supplement: S1 Table — (PDF) [file pgen.1008130.s012.pdf]

Table S1: Pooled exome candidate variants for the NNO1 linkage region

Chr 11 position	Gene	Mutation type	Nucleotide Δ	Protein Δ	CADD score	Cons	gnomAD allele freq
61553534	MYRF	splice acceptor	c.3376-1 G>A	p.Gly1126>Val fs*31	32	High	0/246126
61253294	PPP1R32	missense	c.154 G>A	p.Gly200>Ser	19.01	High	0.24% (669/277018, 10 homozygotes)
60637010	ZP1	missense	c.319 G>A	p.Asn107>Asp	23.1	High	0.2% (629/268740, 4 homozygotes)

Chr 11, chromosome 11; Nucleotide Δ, nucleotide change (cDNA nucleotide change from longest transcript); Protein Δ, predicted protein change; CADD score, combined annotation dependent depletion score (using CADD GRCh37-v1.4); Cons, 100 vertebrate conservation based on UCSC genome browser evaluation; gnomAD allele freq, Allele frequency in gnomAD database with number of alleles and number of homozygotes in parantheses)
