# Supplementary material for: Variants in myelin regulatory factor (MYRF) cause autosomal dominant and syndromic nanophthalmos in humans and retinal degeneration in mice
Source: PLoS Genet. 2019 May 2;15(5):e1008130. doi: 10.1371/journal.pgen.1008130 (PMC6527243; doi:10.1371/journal.pgen.1008130)
Supplement: S2 Table — (PDF) [file pgen.1008130.s013.pdf]

Table S2: MYRF variants in nanophthalmos/high hyperopia probands

SNP ID	Position chr 11	Allele	MYRF Protein change*	SIFT	Conservation$	CADD score	gnomAD # alleles (%)	Cohort freq # alleles (%)
rs149803	61539020	C/G	p.Pro263=	N/A	Moderate	19.93	G=51286 (18.2)	35 (29)
rs75158668	61545842	C/T	Intron Variant	N/A	Low	6.652	T=3726 (1.3)	1 (0.8)
rs61747222	61548796	G/A	p.Arg920His	Tolerated	Low	13.65	A=6546 (2.3)	6 (5)
rs174535	61551356	T/C	p.Ser1051=	N/A	Moderate	12.79	C=102262 (37.5)	30 (25)

SNP ID, Single nucleotide polymorphism ID; * MYRF proteinconsequence from NM_001127392.2 transcript; SIFT, sorting intolerant from tolerant prediction; $ Conservation based on UCSC genome 100 species PhyloP; CADD score, combined annotation dependent depletion score (using CADD GRCh37-v1.4); gnomAD # allele (%), gnomAD allele freq, minor allele number in gnomAD database with allele frequency in paranetheses.
