# Supplementary material for: Variants in myelin regulatory factor (MYRF) cause autosomal dominant and syndromic nanophthalmos in humans and retinal degeneration in mice
Source: PLoS Genet. 2019 May 2;15(5):e1008130. doi: 10.1371/journal.pgen.1008130 (PMC6527243; doi:10.1371/journal.pgen.1008130)
Supplement: S3 Table — (PDF) [file pgen.1008130.s014.pdf]

Table S3: MYRF variants in examined individuals in The Genome Ascertainment Cohort (TGAC)

Transcript consequence*	SNP ID	MYRF Protein change	SIFT	Conservation$	CADD score	gnomAD # alleles / %	Axial length (mm)#
c.989C>G	N/A	p.Pro330Arg	Damaging	High	24.7	G=1 (0.0004%)	23.3 / 23.3
c.880_898del insCTCGAGAGAG	N/A	p.Ser294_Gln300del insLeuGluArgGlu	N/A	High	N/A	N/A	24.2 / 24.1
c.2338G>A	rs201384645	p.Val780Met	Damaging	High	34	A=111 (0.04%)	22.82 / 22.67

* MYRF transcript consequence from NM_001127392.2; SNP ID, Single nucleotide polymorphism ID; SIFT, sorting intolerant from tolerant prediction; $ Conservation based on UCSC genome 100 species PhyloP; CADD score, combined annotation dependent depletion score (using CADD GRCh37-v1.4); gnomAD # allele/ %, gnomAD allele freq, minor allele number in gnomAD database with allele frequency in paranetheses. #, Axial length in right and left eye.
