# Supplementary material for: Variants in myelin regulatory factor (MYRF) cause autosomal dominant and syndromic nanophthalmos in humans and retinal degeneration in mice
Source: PLoS Genet. 2019 May 2;15(5):e1008130. doi: 10.1371/journal.pgen.1008130 (PMC6527243; doi:10.1371/journal.pgen.1008130)
Supplement: S4 Table — (PDF) [file pgen.1008130.s015.pdf]

Table S4: Cell count data from *Myrf* conditional knockout mice.

Genotype		Total area[a] (mm^2)	ONL % of Total Area	INL / Total Area (%)	GCL / Total Area (%)	OS/IS / Total Area (%)	Thickness ONL (µm)	Thickness INL (µm)
Control	Mean	2.5	24	21	19	21	93	85
n=6 anim, 8 eyes, 26 sec	SD	0.2	3	1	2	3	20	30
Rxcre;Myrf$^{+/fl}$	Mean	2.4	23	18**	20	21	81	67
n=3 anim, 6 eyes, 14 sec	SD	0.3	2	1	2	1	10	10
Rxcre;Myrf$^{fl/fl}$	Mean	1.9***	20*	16**	18	12***	61**	50**
n=4 anim, 4 eyes, 15 sec	SD	0.1	2	1	1	2	10	2

Genotype		# cells in ONL	# cells in INL	# cells in GCL	# Cones / Total Retinal Cells	%Cones / Cells ONL	#Rods / Total Retinal Cells	% Rods / Cells ONL
Control	Mean	2337	737	123	109	5	2227	95
	SD	446	95	12	23	1	438	1
Rxcre;Myrf$^{+/fl}$	Mean	2708	658	126	112	4	2509	96
	SD	33	111	37	12	0.5	212	0.5
Rxcre;Myrf$^{fl/fl}$	Mean	1708**	618*	120	55***	3*	1654*	97*
	SD	227	54	3	11	1	216	1

Genotype		ONL #cells/mm^2 retina	INL #cells/mm2 retina	GCL #cells/mm^2
Control	Mean	4805	1746	327
	SD	650	104	56
Rxcre;Myrf$^{+/fl}$	Mean	6152**	1966**	350
	SD	813	136	88
Rxcre;Myrf$^{fl/fl}$	Mean	4993	2259**	386
	SD	198	137	32

[a]Total area refers to total retinal area on the section, including only areas that contain all retinal layers and excluding IS/OS area
[b]ONL-Outer nuclear layer
[c]INL-Inner nuclear layer
[d]GCL-ganglion cell layer
[e]OS/IS-Outer segment plus Inner segment
SD, standard deviation; anim, animals; sec, sections
*** p<0.001, ** p<0.01, * p<0.05 for two-tailed t-test comparison with control
