# Supplementary material for: Variants in myelin regulatory factor (MYRF) cause autosomal dominant and syndromic nanophthalmos in humans and retinal degeneration in mice
Source: PLoS Genet. 2019 May 2;15(5):e1008130. doi: 10.1371/journal.pgen.1008130 (PMC6527243; doi:10.1371/journal.pgen.1008130)
Supplement: S5 Table — (PDF) [file pgen.1008130.s016.pdf]

S5 Table: Electrophysiology data on *Myrf* conditional knockout mice.

Conditions	Stimulus intensity			Control, n= 4 eyes		*Rxcre;Myrf*$^{+/fl}$, n=4 eyes		p value	*Rxcre;Myrf*$^{fl/fl}$, n=12 eyes		p value
				Mean	SD	Mean	SD	wt vs. het	Mean	SD	wt vs. mut
SCOTOPIC	0.01 cd*s/m^2	B	Amplitude (μV)	80	14	80	23	0.98	51	25	0.02*
			Implicit time (ms)	81	1	87	2		85	4	
	10 cd*s/m^2	a	Amplitude (μV)	-89	22	-102	15	0.38	-43	13	0.02*
			Implicit time (ms)	9	0	8	0		9	3	
		B	Amplitude (μV)	134	22	154	31	0.35	80	31	0.006**
			Implicit time (ms)	76	2	77	2		78	4	
	32 cd*s/m^2	a	Amplitude (μV)	-106	26	-122	16	0.35	-48	13	0.02*
			Implicit time (ms)	6.9	0.2	6.9	0.3		6.9	0.4	
		B	Amplitude (μV)	151	33	177	33	0.31	83	32	0.02*
			Implicit time (ms)	71	3	74	1		76	4	
PHOTOPIC	10 cd*s/m^2	B	Amplitude (μV)	48	3	54	4	0.04*	20	9	3x10^{-7} ***
			Implicit time (ms)	40	2	39	1		46	4	
	32 cd*s/m^2	B	Amplitude (μV)	52	5	56	4	0.38	23	9	9x10^{-6} ***
			Implicit time (ms)	39	2	38	1		44	4	
	100 cd*s/m^2	B	Amplitude (μV)	53	7	59	6	0.23	25	9	0.0004***
			Implicit time (ms)	39	2	38	1		43	3	
FLICKER	20 cd*s/m^2	P1	Amplitude (μV)	30.75	0.86	35.96	3.39	0.05	17.53	6.35	1x10^{-5} ***
			Implicit time (ms)	49.00	1.73	47.13	0.63		51.5	2.71	

a, a-wave; B, B-wave; P1, peak flicker amplitude; SD, standard deviation; *** p<0.001; ** p<0.01; * p<0.05
