# Supplementary material for: Variants in myelin regulatory factor (MYRF) cause autosomal dominant and syndromic nanophthalmos in humans and retinal degeneration in mice
Source: PLoS Genet. 2019 May 2;15(5):e1008130. doi: 10.1371/journal.pgen.1008130 (PMC6527243; doi:10.1371/journal.pgen.1008130)
Supplement: S6 Table — (PDF) [file pgen.1008130.s017.pdf]

Table S6: MYRF screening primers and conditions

Oligo Name	Oligo Sequence	Amplicon Size	Annealing Temp*
MYRF Exon 1A for	ACCGTAGCCGGAGCCCA	250bp	60
MYRF Exon 1A rev	CGGAAACACAGCGAGGAG		
MYRF Exon 1B for	GGGAAAGGGTTTTACCGAAG	501bp	55
MYRF Exon 1B rev	TGCAGGTGAGGAAAAAGGAC		
MYRF Exon 2/3 for	AGACTCAAGGTGGCCTTGTG	1165bp	55
MYRF Exon 2/3 rev	GGTATGCAGGTGTTCCCATG		
MYRF Exon 2 rev seq	GGGAACTATGGAGATTTCGG		
MYRF Exon 3 for seq	CCGAAATCTCCATAGTTCCC		
MYRF Exon 2/3 for-2	CTGTACCCAGATCCATGCCC	904 bp	55
MYRF Exon 2/3 rev-2	TATGCCGCTGTATCCCCCTA		
MYRF Exon 4 for	TGTGACGAAACCTCAGTGTC	316bp	55
MYRF Exon 4 rev	TGACCAGCGATAATCTCAGC		
MYRF Exon 5 for	TCAGGACGGGGTGGAAGCA	487bp	60
MYRF Exon 5 rev	TGCATCCCGGTCCCTACCT		
MYRF Exon 5 for-2	TAGTAGGTGGCTGACCAACG		55
MYRF Exon 5 rev-2	GGCCTTCATGTACAGATCCCG		
MYRF Exon 6/7 for	TGACCTTGAGTGTGACACTG	909bp	56
MYRF Exon 6/7 rev	TGTGAAATGAGTGCTCCCAG		
MYRF Exon 8 for	AGCCCTGTACTAGAATCCTC	452bp	55
MYRF Exon 8 rev	TTTCTGGGGTCTTTCAGTGC		
MYRF Exon 9/10 for	TTCCTGGTGAGCTCTAGTTG	772bp	55
MYRF Exon 9/10 rev	CTGGCCAGATGCATGTTCAT		
MYRF Exon 11 for	GACTTAAGGATGGGAAGAGC	572bp	55
MYRF Exon 11 rev	CAAGCCCAACTTGAACTGTC		
MYRF Exon 11 for-2	ACAGGCTGAGCCATTTCACA	621bp	55
MYRF Exon 11 rev-2	GCCCAACTTGAACTGTCCCT		
MYRF Exon 12/13 for	GACAGTTCAAGTTGGGCTTG	810bp	55
MYRF Exon 12/13 rev	GTAGGATTTCCAGGTGACAG		
MYRF Exon 12 for	AGGGACAGTTCAAGTTGGGC	489bp	55
MYRF Exon 12 rev	TAGATCGGGATTCGAGGCCA		
MYRF Exon 13 for	GCCTCGAATCCCGATCTAACC	362bp	55
MYRF Exon 13 rev	AGTTCCGGGAGTCAAGAGGA		
MYRF Exon 14 for	ATCCTGTGAACACTGGTTCG	462bp	55
MYRF Exon 14 rev	ATCTCCTTGAACACTCACCC		
MYRF Exon 15/16 for	TGACATCTGAGACACCAGTC	800bp	55
MYRF Exon 15/16 rev	AAGAAAGAGAAACGGGGCTG		
MYRF Exon 17/18 for	CTTTCTGGCTTGCAGTTCTC	1033bp	55
MYRF Exon 17/18 rev	AGAAGACACAGTCAGAGGTGG		
MYRF Exon 17 for	CCAGCCCCGTTTCTCTTTCT	481bp	55
MYRF Exon 17 rev	TCCAGTGCCCTCTCTCTAGG		
MYRF Exon 18 for	CTCAGGAGCAAGGACCACAG	236bp	55
MYRF Exon 18 rev	CTCATGGCTCTCTGACCTGG		
MYRF Exon 19/22 for	CTGTCTGGCCTGTTTAGTGC	1440bp	55
MYRF Exon 19/22 rev	ACTGACAAGTCTGAACCCAG		
MYRF Exon 21/22 for seq	CATACAGCCCTCTTTGCTGC		
MYRF Exon 19/20 rev seq	AACATGGCCAGGGTTAAAGC		
MYRF Exon 19/20 for	GGGTTGGAACATGGGGTAGG	573bp	57
MYRF Exon 19/20 rev	CCAGAGGCTGTATGAGCCAG		
MYRF Exon 21 for	GGGGCTTCTCTGGCTCATAC	459bp	55
MYRF Exon 21 rev	AGGCACAGGCTAAGAAGCTG		
MYRF Exon 22 for	GGACCAAGAGACACATGGGG	515bp	55
MYRF Exon 22 rev	CTGCAGGCCTGATTCCTGAT		
MYRF Exon 23/25 for	AAGGTGTGAGTGACTGCTTC	1083bp	55
MYRF Exon 23/25 rev	CTATAACTCCCAGCTTCTGC		
MYRF Exon 24/25 for seq	CTTGGACACTGTCTCTTCTG		
MYRF Exon 23 rev seq	CAGAAGAGACAGTGTCCAAG		
MYRF Exon 23/24 for	CCTTACTCCTGCCCCAACAG	623bp	55
MYRF Exon 23/24 rev	GGCCAGGTCATTTAGCCAGT		
MYRF Exon 25 for	CAGGCTGGGTGGAGATTCAG	412bp	55
MYRF Exon 25 rev	TGCTCAGCAGAGTGCAATGA		
MYRF Exon 26/27 for	GAACATGCTCCTTCCTATCTC	579bp	55
MYRF Exon 26/27 rev	CTCCAGTGTAACACCATTGC		

* PCR Conditions: 95°C 10 min, 35 cycles: 95°C 30sec, Annealing Temp 30 sec, 72° C 1 min; 72°C 10 min
