# Supplementary material for: Variants in myelin regulatory factor (MYRF) cause autosomal dominant and syndromic nanophthalmos in humans and retinal degeneration in mice
Source: PLoS Genet. 2019 May 2;15(5):e1008130. doi: 10.1371/journal.pgen.1008130 (PMC6527243; doi:10.1371/journal.pgen.1008130)
Supplement: S7 Table — (PDF) [file pgen.1008130.s018.pdf]

Table S7. Other primers and PCR conditions used in this study.

Experiment	Oligo Sequence	Amplicon Size	Annealing Temp
PPP1R32 exon 6 (rs148713373)	for 5'-CTTCCCATCTTCCAATCCAC-3'	623bp	55°C
	rev 5'-GCAGTGGTTTTCACCTAAGC-3'		
ZP1 exon 3 (rs145707301)	for 5'-GAACCAGAAAGGAGCCAAAG-3'	642bp	55°C
	rev 5'-GCCCAGTAAAAGGCTGTTAG-3'		
MYRF mini-gene cloning	for 5'-ATATATATCGATCATCCTGTCCTTCCGTGAAT-3'	2903bp	55°C
	rev 5'- ATATATCTCGAGTCCACTCCTAGCCCCAAT-3'		
MYRF splicing evaluation	for 5'-CATCCTGTCCTTCCGTGAAT-3'	698 genomic, 510 spliced	55°C
	rev 5'-GTCTCAAAAGGGGAGGCAAG-3'		
Rxcre mouse genotyping	for 5'-GCCTGCCCTCCCTGTGGATGCCACCT-3'	357bp	60°C
	rev 5'-GCCAGGGCCTGCTTGGCTCTCTCCCC-3		
Myrf floxed genotyping	for 5'-AGGAGTGTTGTGGGAAGTGG-3'	wt-281bp, flox 489bp	60°C
	rev 5'-CCCAGGCTGAAGATGGAATA-3'		

wt, wild-type MYRF allele; flox, MYRF floxed allele
